# Does resistance training make a difference to the quality of life or heart health for older adults compared to aerobic exercise? A systematic review protocol from The People’s Review

**DOI:** 10.1371/journal.pone.0337017

**Published:** 2026-06-10

**Authors:** Éle Quinn, Laura Bosner, Patricia Logullo, Kevin Murray, KM. Saif-Ur-Rahman, Charlene Young, Derek Stewart, Maureen Smith, Jeremy Holt, Shaun Treweek, Chris Noone, David Moher, Sinéad M. Hynes, The People

**Affiliations:** 1 Discipline of Occupational Therapy, School of Health Sciences, University of Galway, Galway, Ireland; 2 Evidence Synthesis Ireland and Cochrane Ireland, University of Galway, Galway, Ireland; 3 Centre for Health Research Methodology, School of Nursing and Midwifery, University of Galway, Galway, Ireland; 4 School of Medicine, University of Galway, Galway, Ireland; 5 Independent Researcher, United Kingdom of Great Britain and Northern Ireland; 6 School of Pharmacy and Medical Sciences, University of Galway, Galway, Ireland; 7 Public Partner, United Kingdom of Great Britain and Northern Ireland; 8 HRB Trials Methodology Research Network, University of Galway, Galway, Ireland; 9 Aberdeen Centre for Evaluation, University of Aberdeen, United Kingdom; 10 School of Psychology, University of Galway, Galway, Ireland; 11 Centre for Journalology, Methodological and Implementation Research, Ottawa Hospital Research Institute, Ottawa, Canada; University of Glasgow School of Health and Wellbeing, UNITED KINGDOM OF GREAT BRITAIN AND NORTHERN IRELAND

## Abstract

**Background:**

Systematic reviews bring together all the evidence on a health topic in an organised and careful way. The People’s Review aims to help the public understand what systematic reviews are and why they matter by designing and conducting their own systematic review. The question chosen for The People’s Review is: *Does resistance training make a difference to quality of life and/or heart health for older adults compared to aerobic exercise?* This paper outlines how we will carry out this review.

**Methods:**

This is a systematic review involving the public throughout. This review will search for, include and summarise: randomised controlled trials, with older adults (50 + years), that compare resistance training (such as lifting weights) with aerobic exercise (such as walking or running), and measure quality of life or heart health. First, the technical team will search research databases to find possible studies. The public will look at summaries of these to find studies that might be relevant. Then, two members of the technical team will read the full studies and decide which ones to include. Next, the public will help collect some of the key information from the included studies. The technical team will record the rest. The public and the technical team will work together to check for biases (or flaws in how the studies were done) in the studies. Finally, if possible, the team will combine the study results using a method called meta-analysis (a way of pooling numbers together). If we can’t combine the numbers, we will write a summary of what the studies found

**Discussion:**

This review will summarise all the available evidence that addresses the review question. This review could support the public to make decisions about what type of exercise to engage in as they age, and influence exercise guidelines, clinical practice and future research.

## Introduction

This is a systematic review protocol from The People’s Review – an online, novel citizen science project involving members of the public in a systematic review. The overall aim of The People’s Review is to support the public in understanding of what systematic reviews are and why they matter [1]. With this in mind, this protocol is written in plain English to make it accessible to as many people as possible.

This protocol was produced with 366 members of the public, cited in this protocol as “*the People*”. *Stage 1: Suggest a Question* of The People’s Review opened on 22^nd^ of April 2025 and *the People* joined us in developing this protocol through *Stage 1, Stage 2: Choose the Question and Stage 3: Plan the Review*. The remaining stages of The People’s Review will involve the public in tasks related to producing the systematic review itself and will be open between September 2025 – April 2026.

There is also a group of researchers and patient and public involvement (PPI) contributors working behind the scenes. Throughout this protocol, we refer to this group as the “*Technical Team*”. We are all one review team who have created this protocol together and will produce the corresponding systematic review together. We make the distinction between *the People* and *the* Technical Team throughout this protocol so it is clear and transparent who is doing what parts of the review.

### What this review is about: the importance of exercising

As we age, our bodies change. Ageing gradually impacts our heart health and fitness. A decline in heart health is closely linked to the onset of diseases, including heart failure [2], diabetes [3], and the narrowing of arteries [4,5]. Therefore, it is important to find the best ways to support healthy ageing in older adults, slowing the decline of heart health, preventing the onset of diseases, and ultimately leading to a better quality of life [6].

Regular exercise has a positive impact on healthy ageing [7,8]. Exercise prevents the onset of disease, maintains function, improves mental health, and brings social benefits. In particular, regular exercise supports heart health for older adults [9–11].

### Types of exercise we will investigate

Different types of exercise are promoted to improve heart health, quality of life, muscle strength, mobility and many other health outcomes [10–12]. Aerobic exercise is an activity that increases the heart rate and breathing, while engaging large muscle groups over a sustained period. These exercises improve heart health, fitness and endurance. Examples include brisk walking, running, swimming, cycling, or dancing. Resistance training is another form of exercise that involves using external force or load to challenge and strengthen muscles. The external force could be lifting weights, pulling against elastic resistance bands, or using one’s own body weight to work against gravity.

Traditionally, aerobic training was recommended to improve heart health [10,12,13]. However, in recent years, emerging research suggests that resistance training may also play an important role in improving heart health [14–17]. Although the main goal of resistance training is to increase muscle strength [18], the American Heart Association recommends engaging in regular resistance training to lower the risk of heart disease and maintain a healthy heart [19].

However, there is still uncertainty about how resistance and aerobic training compare to each other for supporting heart health [20] and quality of life [21]. A recent systematic review explored the effects of aerobic versus resistance training on heart health in older adults [22]. However, that review did not focus on quality of life, and had several limitations. Therefore, it is still unclear whether resistance training makes a difference to the quality of life and heart health for older adults compared to aerobic exercise. This review will compare these two types of exercise: resistance and aerobic training.

### How resistance training might improve heart health and quality of life

Resistance training causes a person’s blood pressure to lower, meaning there is less pressure put on the heart as it pumps blood around the body [15,16]. There is also an increase in the amount of blood the heart can hold and pump out with each beat, meaning the heart works more efficiently [17]. Resistance training improves how muscles and blood vessels use and transport oxygen, so the heart doesn’t have to work as hard [23–25]. It can also lower cholesterol, balance blood sugars and reduce body fat, which may impact heart health [14,26]. Through these effects, resistance training has been shown to reduce the risk of developing heart disease [19].

Resistance training may improve more than just heart health for older adults. It can also enhance overall physical health [27,28], lower the risks of falls [29], and improve mental health in older adults [21,30–32]. Any type of physical activity can improve quality of life [33], including resistance training, probably due to the release of feel-good chemicals in the brain and strengthening pathways in the brain that control emotions and mood [31,32,34]. Additionally, resistance training can help older adult’s brain function [35] such as concentration and memory.

### Why it is important to do this systematic review

Finding out whether resistance training is equally good or better than aerobic exercise may be useful to the public, healthcare workers (such as doctors, nurses, or physiotherapists), policymakers, and researchers to inform recommendations about what older people can do to support their physical and mental well-being.

Perhaps the most important reason for conducting this systematic review is that the question was selected by the public. Through three rounds of priority-setting surveys, *the People* decided the final question for this review.

### What is the aim of this review?

This systematic review aims to find out if resistance training makes a difference to quality of life and/or heart health for older adults (aged 50 years or older) compared to aerobic exercise.

## Methods

**Fig 1:**
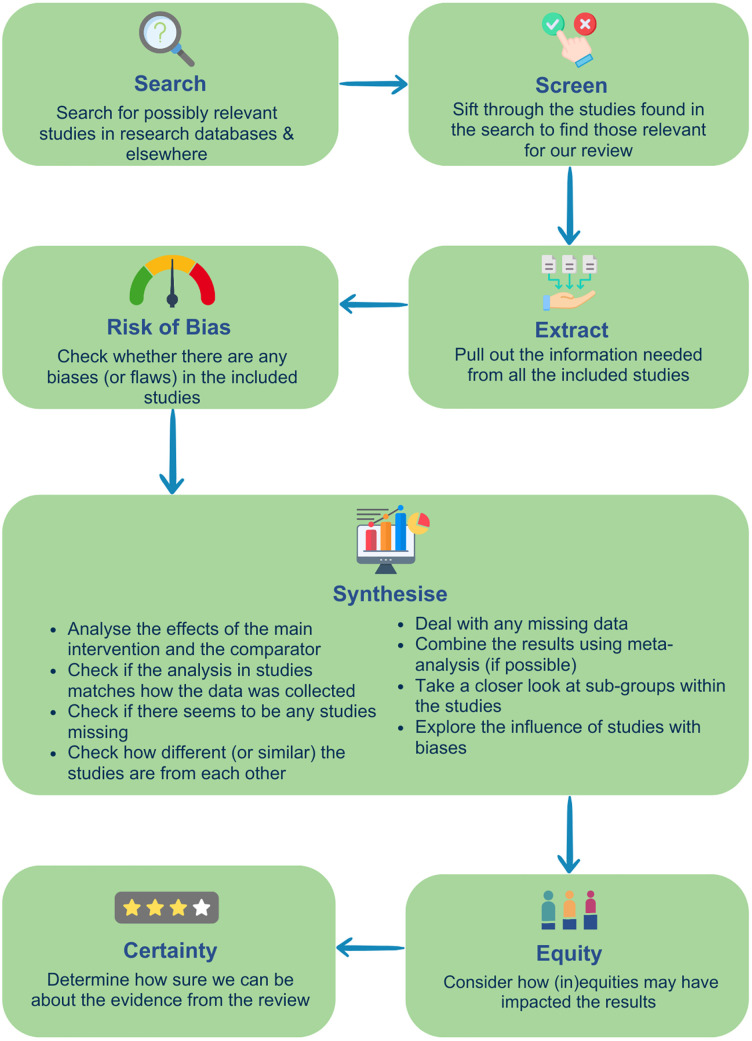
A flow diagram of this systematic review’s methods.

This is a systematic review protocol with public involvement throughout. This protocol follows the PRISMA-P reporting checklist [36] to ensure it is clear, complete and easy for everyone to understand. The protocol is registered in PROSPERO: CRD420251156252. See Fig 1 for an overview of the methods.

**What is a systematic review protocol?**

A systematic review protocol is a plan for how a systematic review will be done. A protocol should be written up and shared *before* the authors of the review begin searching for and analysing the studies to be included in the review. A systematic review protocol improves the quality of the review, holds authors accountable to the set out plan, and allows others to take a look at the work before it starts [37].

Results for the review have not yet been generated. Review results are expected to be completed by August 2026. Ethics approval is not required for systematic reviews. However, we have received ethical approval from the University of Galway Research Ethics Committee (2023.06.012) to involve the public in The People’s Review.

### How will *the People* be supported to be involved in doing this review?

The People’s Review follows the principles of ‘learning by doing’ [38] and citizen science [39]. There are many tasks involved in a systematic review. To support *the People* to get involved in these tasks we will provide bespoke interactive training for each systematic review task including animations, explanations of key concepts, and practice material. Support will also be provided in real time during the task through pop-out reference material and hint features. As with all citizen science projects there are several quality control layers to ensure our review is accurate and high-quality including agreement algorithms and verification processes. We will provide regular updates via email, social media, newsletters and on our website to further support learning and maintain an open and honest feedback loop. Further description of processes to support involved in The People’s Review are described elsewhere [1].

### What studies will we include in our review? – the eligibility criteria

A study will be included in this systematic review if it:

1. Is a randomised controlled trial (RCT)
2. Includes older adults
3. Compares resistance training with aerobic exercise
4. And measures quality of life and/or heart health

The study must meet all four of these criteria for it to be included in the review, which are described in further detail below.

### Randomised controlled trials (RCTs) only – the study design

We will only include randomised controlled trials (RCTs) in our review. Randomised trials are widely considered the most reliable way to evaluate whether healthcare interventions work or not. We will include all types of randomised trials, including simple designs (like those with two or more groups) and more sophisticated designs, such as cluster-randomised trials (where participants are randomised in a cluster or group, such as by clinic or nursing home unit) and cross-over trials (where participants are randomly assigned to either the resistance training or aerobic exercise group and then switched). All other types of studies will be excluded from our review.

### Older adults – the population

We define older adults as people over the age of 50. Trials with people aged 49 years or younger will be excluded from our review. If a trial includes both people over 50 and under 50, we will include it only if the data for people over 50 is reported separately. Trials where the data for people over 50 and under 50 is not reported separately will be excluded.

There is some disagreement in the literature about what we mean by an ‘older adult’ [40,41]. For example, the World Health Organization defines older adults as people over the age of 60 [6]. Others argue that older adults include people over the age of 50, especially where life expectancy is lower [42,43]. For this review, *the People* decided to focus on adults over the age of 50.

### Resistance training and aerobic exercise – the intervention and comparator

We will include trials that compare resistance training with aerobic exercise. For our review, we consider resistance training to be the main intervention being studied and aerobic exercise to be the comparator. We define resistance training as a form of exercise that involves using external force or load to challenge and strengthen your muscles. The external force could be lifting weights, pulling against elastic resistance bands, lifting weighted household objects (such as bricks or bottles of water), or using your own body weight (such as squats, or push-ups).

We define aerobic exercise as any activity that increases heart rate and breathing, while engaging large muscle groups over a sustained period. These exercises improve heart health, fitness and endurance. Examples include brisk walking, running, swimming, cycling, dancing, or sports. *The People* decided these definitions of resistance training and aerobic exercise.

We will include trials that compare resistance training with aerobic exercise where the programme includes at least eight sessions, regardless of how often (frequency) and how long (duration) each session was. *The People* chose this threshold because it provides enough time for participants to receive enough exercise for their bodies to adapt and show changes in fitness, while still being feasible to carry out in real-world settings. Trials with fewer than eight sessions will be excluded from this review.

### Quality of life and/or heart health – the outcomes

We are interested in two outcomes for this review: quality of life and heart health. Therefore, we will include trials that measured either quality of life or heart health. Quality of life refers to a person’s overall satisfaction with life, involving the interaction between physical health, emotional well-being, independence, social relationships, and the environment. We will use the World Health Organisation’s definition of quality of life as “individuals’ perceptions of their position in life in the context of the culture and value systems in which they live and in relation to their goals, expectations, standards and concerns” [44].

Quality of life is usually measured using specially designed questionnaires. Some commonly used quality of life questionnaires include the Short-Form 36 [45,46], EuroQol [47], and the WHOQOL [48]. These measures often include different areas including both the mental and physical aspects of quality of life. Both mental and physical health aspects will be collected and analysed separately, if available.

There are many outcomes we could use to measure heart health – blood pressure, resting heart rate, fat (or lipid) markers such as cholesterol, VO₂max (the maximum amount of oxygen the body can use during exercise) and many more. However, for this review we will use functional fitness as the measure for heart health. *The People* prioritised several heart health outcomes and selected functional fitness as the outcome to focus on. Functional fitness (or functional aerobic capacity) is a person’s ability to keep exercising over a sustained time period, at a level where they can still talk without becoming very breathless. It is one of the best measures of overall fitness and heart health as it evaluates all the body’s systems working together during exercise [49,50]. While functional fitness does not directly measure heart function through biological markers like blood pressure and cholesterol levels, it is a valuable measure of overall heart health. We will include studies that measure functional fitness through accepted tests such as the 6-minute walk test (6MWT), shuttle tests or other methods.

No other outcomes will be explored in this review. We acknowledge that this review could focus on many other outcomes related to heart health. For this review we are focusing on functional fitness only as the measure of heart health. *The People* decided to focus only on functional fitness, as this was considered the most important measure of heart health for them.

### How will we search for the studies for our review? – the search strategy

#### Searching for the studies in electronic databases.

We will develop a search strategy with the help of a medical librarian and following best practice for developing, testing and reporting search strategies [51,52]. The search strategy will be independently peer-reviewed by a medical librarian using the PRESS checklist [52].

To search for relevant studies, we will combine three concepts:

- The population - ‘older adults’ (using a pre-tested filter for people over the age of 50 [53]).
- The intervention - ‘resistance training’
- The comparator – ‘aerobic exercise’
- The study design – ‘randomised controlled trials’ (using the Cochrane filter [54]).

For each concept, we will search for different words and subject headings and combine them together. For further details of our search strategy, please see Appendix 1.

We will search online databases that contain millions of studies from around the world. To make sure we capture the breadth of research, we will search the following databases:

- MEDLINE Ovid
- Embase Ovid
- PsycINFO
- CINAHL
- PEDro
- LILACS
- Cochrane Central Register of Controlled Trials (CENTRAL)

We will not limit the search by language of publication or publication date.

### Searching for the studies in other places

It is not enough to only search online databases of finished and published trials, as some trials could be missed. Therefore, we will search for ongoing trials on the US National Institutes of Health Ongoing Trials Register ClinicalTrials.gov (https://www.clinicaltrials.gov) and the World Health Organisation International Clinical Trials Registry Platform (ICTRP; https://www.who.int/tools/clinical-trials-registry-platform).

Sometimes studies may be missed because they are not published in journals. For example, there could be valuable information in government reports, conference presentations, pre-print servers, theses or dissertations. This is known as grey literature. To try and find grey literature, we will search ProQuest Dissertations and Theses Citation Index via the Web of Science and the Data Archiving and Networked Services (DANS) Data Station, which allows access to all records in greynet.org and all other records previously hosted on the System for Information on Grey Literature database. We will also search medRXiv, the pre-print server for health sciences (https://www.medrxiv.org/).

Additionally, we will look at the reference lists of the included studies to find trials that may not be brought up by the database searches. We will also see which newer studies have cited the included studies. Finally, we will check the reference lists of similar systematic reviews [22,30] published on this topic also.

### How will we select the studies for our review? – screening

As we will search across multiple databases, some studies will likely be included more than once, and we will need to remove these duplicates. The *Technical Team* will use Rayyan [55] to remove the duplicates.

We will then select the studies for our review in two steps, in a process known as screening. We will report the process on how we selected the studies using a flow diagram [36], clearly outlining the number of studies that made it through each round.

#### Step 1: Title and abstract screening.

In the first round of the screening process, *the* *People* will decide whether studies are relevant for our review by looking at the titles and abstracts of potential studies and deciding if they are ‘Possibly relevant’ or ‘Not relevant’. Each abstract will be screened by at least four people. If there is a discrepancy, a member of the *Technical Team* will act as a resolver to make the final decision about whether it should make it through the next round of screening, full-text screening. This process has been previously described elsewhere [1,56,57].

#### Step 2: Full-text screening.

Two members of the *Technical Team* will read the full texts of all abstracts that *the People* decided were possibly relevant for this review. Reading the complete versions of the studies will allow the team to confirm whether they should be included in the review or not. If these two members have different opinions about a study, they will decide whether it should (or should not) be included through discussion or by consulting a third person, if they don’t agree.

### How will we collect the information from the studies? – data extraction

*The People* will extract some pieces of information from the studies, and the *Technical Team* will extract others. *The People* will look for information about the participants (for example, number of people who took part in the trial, sex, disability, location of the study), the intervention/comparator (for example, a description of the exercise and comparator) and outcomes (what was measured and how). The *Technical Team* will extract all other information, including results data.

*The People* can extract information from as many studies as they wish, but we ask that they extract information at least one study each. Each study will have its data extracted by at least four different members of the public. The remaining data, mainly about the trial results, will be collected from the studies by two independent members of the *Technical* *Team*. For both *the People* and the *Technical Team*, the data will be collected from the studies using a data extraction form that will be designed for this review, pilot-tested, and implemented online.

People may extract slightly different information from each study. When there is less than 80% agreement for each data item extracted by *the People*, a resolver from the *Technical Team* will make the final decision. If the *Technical Team* members do not agree, this disagreement will be resolved through discussion or involving a third team member if necessary.

If reported in the studies, the following information will be extracted (between *the People* and the *Technical Team*). This will include information about:

- the study itself, including: the study/report ID, year of publication, journal, authors and funding
- the participants in the trial, including: age, sex, gender, sexual identity, race, ethnicity or ancestry, socio-economic status, level of education, disability, and residence (country and type, if rural/urban).
- the intervention groups: description of both the resistance and aerobic trainings provided, where training took place, who delivered or supervised the training, exercising duration (how long) and frequency (how many).
- the outcomes: quality of life or functional fitness, how these outcomes were measured and defined, and the units of measurement.
- the results, including: the number of participants allocated to the intervention and the comparison groups, number of participants included in the analysis.

### How will we check for bias in the studies? – risk of bias

An important step in a systematic review is checking if there are any potential flaws (known as bias) in how the studies were done, how these flaws may have impacted the results, and therefore influence how much or how little we can trust the study. We call this risk of bias. To measure the trial’s risk of bias, we will use Cochrane’s original risk of bias tool [58]. This tool looks at seven different aspects (called domains) of the randomised trial process for each outcome (i.e., quality of life or functional fitness) within the trial. While there has been an update to the Cochrane risk of bias assessment (ROB-2) tool [59] we will use the original version as it is easier to use than ROB-2 [60–63]. The domains of the risk of bias tool are described in Table 1.

**Table 1:** What do we look at when checking for the risk of bias in trials.

Domain	What it is	Why it matters	Timing
**Random sequence generation**	The way researchers ensure the assignment of participants to the intervention and comparison groups happen at random. For example, by flipping a coin, rolling a dice, or using a computer programme.	Randomising participants to each group makes sure the researchers do not choose who goes into what group. It also ensures the groups are similar from the beginning and the distribution is fair (for example, with groups that are roughly the same size and with similar characteristics).	At the beginning of the trial before participants are being placed into the intervention or comparison group.
**Allocation concealment**	Making sure the people enrolling participants do not know or cannot predict which group the participant will be assigned to. This domain is about the method used to hide the allocation.	It is important that the people running the trial are not able to influence which treatment each participant gets. For example, if the person enrolling a participant knows that the next assignment is to intervention group (consciously or unconsciously) assign a participant they think will do better in the intervention group.	At the beginning of the trial, before and during the randomisation process.
**Blinding of participants and personnel**	Making sure that both the participants receiving either the intervention or comparator (the participants), and the people running the trial (the personnel) are unaware of which group participants are in.	Participants and/or the people running the trial might act differently if they know whether participants are getting the intervention or comparison.	During the trial when the participants are receiving the intervention or comparison.
**Blinding of outcome assessors**	Making sure that the people measuring the outcomes are unaware which group participants are in.	If the outcome assessor knows whether the participant is in the intervention or comparison group, they might change the outcome to make the results more favourable.	When the outcomes are measured (usually before and after the intervention)
**Incomplete outcome data**	When there is missing data because participants may have dropped out of the trial or did not complete all of the outcome measures. This domain looks at how much data is missing and if it was handled properly.	It is normal to have some missing information in trials. But if there is lots of missing information especially in one group and not another, it can influence the trial results.	When the results of the trial are being analysed.
**Selective reporting**	Whether the researchers reported all the results they planned to in the protocol.	Leaving out results that researchers didn’t like can give a false impression of how well something worked.	Happens when the results of the trial are being written up and published.
**Other bias**	This is a place to note any other problems that may have influenced the results of the trial; for example, conflicts of interest, not following the plan, or stopping the trial early.	Sometimes there are problems that don’t fit neatly into the other domains.	At any stage during the trial.

For each domain, we will assign a judgement of either low, high or unclear risk of bias. As with previous stages, bespoke training will be available to explain key concepts about risk of bias, provide worked examples and allow people to practice before assessing the risk of bias in the included studies. The public can assess the risk of bias in as many studies as they choose; however, they will be asked to assess the risk of bias in at least one study.

*The People* will look at the first four risk of bias domains, with the remaining three domains to be assessed independently by two members of the *Technical Team*. Each study will be assessed by at least four different members of the public. If there is less than 80% agreement for each domain assessed by *the People*, a member of the *Technical Team* will act as a resolver to make the final decision. If discrepancies arise in the *Technical Team*, they will resolve it through discussion or with a third assessor.

If cluster randomised trials are included, the *Technical Team* will assess all seven risk of bias domains for these trials. This is because cluster randomised trials require additional considerations, such as whether the analysis accounts for clusters, whether effects are overestimated, or if there were recruitment issues [64,65].

The results from the assessment and the *Technical Team’s* assessment will be combined and summarised in tables and graphs. We will also summarise the risk of bias within each study in a table.

### How will we analyse and summarise the results from each trial?

Once we have extracted the data from the included trials and assessed them for risk of bias, we will then synthesise the results. Synthesising means carefully combining the results data from all the individual trials to provide an overall picture of the evidence.

**Fig 2:**
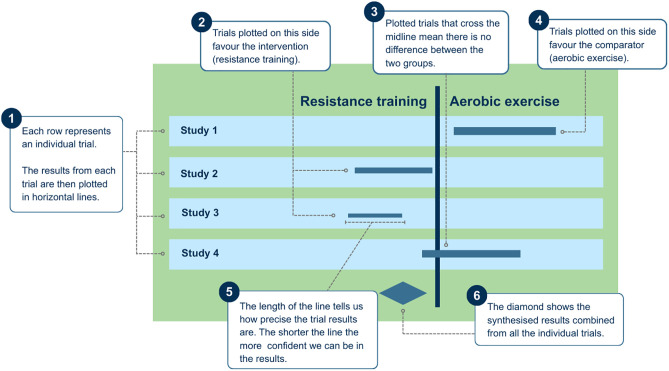
What is a forest plot?.

In systematic reviews that compare two treatments (like this one), we typically use a statistical method known as meta-analysis to synthesise numerical data. Meta-analysis combines the results data from each individual trial and produces a summary, or overall result. The result of synthesising the data using meta-analysis produces a graph called a forest plot (described in Fig 2). Sometimes it is not always appropriate or possible to do a meta-analysis and produce a forest plot. In this case, we will use other ways [65] to summarise the results, including words and tables.

### Measuring the effects of the intervention versus the comparator

We want to find out how resistance training compares to aerobic exercise on quality of life and heart health (measured by functional fitness). To do this, we must calculate the difference between the two groups. What calculation we use is based on two factors: 1) the type of data we are working with, and 2) whether the studies measure the outcomes in the same way.

The type of data for both of our outcomes, quality of life and functional fitness, is continuous. Continuous outcomes are based on scales with values that are in a specified range – everyday examples of continuous data include height, weight, and temperature.

The *Technical Team* will calculate either the mean difference or the standardised mean difference to measure the difference between the two groups in each study. If the outcomes are measured in the same way in all trials, using the same test, we will calculate the mean difference and 95% confidence interval. Mean difference is the average difference between the two groups.

**How to calculate mean difference – a worked example**

Say people doing resistance training rate their quality of life as 70 out of 100. People who do aerobic exercise rate it as 65 out of 100. The mean difference would then be 5. A 95% confidence interval is the range of numbers that is likely to include the true mean difference. For example, if resistance training improves quality of life by 5 points more than aerobic exercise, with a confidence interval of 3–7 points, this suggests the real difference is likely between 3 and 7. The 95% confidence interval means that if we repeated the study 100 times, in 95 of those studies, the range we would find (like 3–7 points) would include the true mean difference.

If the outcomes are not measured in the same way, using the same scale (which is quite likely for both quality of life and functional fitness), we will calculate the standardised mean difference. For example, one study might score quality of life out of 10, and another out of 100. The standardised mean difference puts all the results on the same scale, so we can still compare them fairly.

### How will we account for problems with study designs that may affect the results? – unit of analysis issues

Data collected in randomised trials should be analysed in ways that match how the data was collected. For simple trials where each participant is randomly allocated to one of two groups, there are usually no issues. However, for more sophisticated trial designs there are some considerations that need to be made, to ensure that we do not over or underestimate the effects of the intervention. These considerations are described in Table 2.

**Table 2:** Study designs that need to be accounted for in this review.

Randomised trial designs	Explained	What we will do about these designs in this review
Randomised trials with more than two groups	For example, one group of participants could do resistance training, another could do aerobic exercise, a third could do both resistance training and aerobic exercise.	We will extract data only from the resistance training group and the aerobic exercise group. We will not analyse data from combined interventions (for example interventions that use aerobic and resistance training).
Crossover trials	Where participants are randomly assigned to either resistance training (the intervention) or aerobic exercise (the comparator). Then the groups swap and do the other exercise type. However, the effects of the first type of exercise might still be there when people start the second type. That makes it harder to tell which exercise is really causing the results.	To prevent the impact of the carryover effect we will use only the results from the first period before the groups swap.
Cluster randomised trials	When participants are randomised in a cluster or group, such as by clinic, school or nursing home.	The *Technical Team* will assess if the authors of the cluster trials have appropriately accounted for clustering and get advice from a statistician if needed. If the authors have not appropriately accounted for clusters, we will adjust for this using methods recommended in the Cochrane handbook. These methods require specific data (such as the intracluster correlation coefficient (ICC)) to make appropriate adjustments. If this is not reported in the study we will contact the study authors or use external estimates and clearly document the source used [66].

### What will we do if there is information missing that we need? – dealing with missing data

It is common for trials not to report all the information needed for a review. When this happens, the *Technical Team* will initially contact the trial authors to request the missing information. We will record the amount of missing data, specify what data is missing, and the reasons for missing data (if known). If the authors have filled in missing data by using estimated guesses (known as imputation), we’ll decide if the approach is appropriate based on best practice [67].

If the authors do not respond to us with the missing data, the *Technical Team* may use accepted methods to estimate the missing values (known as imputation). For example, if standard deviations (which show how spread out the results are from the average) are missing and cannot be calculated from the data, the *Technical Team* will follow Furukawa’s guidance on imputing this data [68]. We will only fill in missing values if there is a small amount (less than 10%) of data missing. If there are large amounts of missing data in a study, we will exclude it the from the meta-analysis (the statistical method of combining the results). We will, however, include the study in our review and summarise it in writing. We will make note of any missing data and discuss the potential impact of this on the results of our review in the discussion section.

### How will we assess whether the studies are similar or different to each other? - heterogeneity

Heterogeneity is the extent to which the trials are different from each other. It is important to check how different the trials are so we have a clear picture of the evidence. There are also practical reasons for the review because if they vary too much, combining the results in a meta-analysis (shown in a forest plot) could give misleading results. In systematic reviews, heterogeneity can be measured in several ways. Each test is different and has strengths and weaknesses. We will use four different tests to account for these limitations. The *Technical Team* will conduct all four tests, and *the People* will help interpret the results of the tests.

The first test involves visually inspecting the forest plot (graph) produced from the meta-analysis. We will look out for:

1. The point estimate distribution
2. Overlapping confidence intervals
3. The width of the confidence intervals

**Fig 3:**
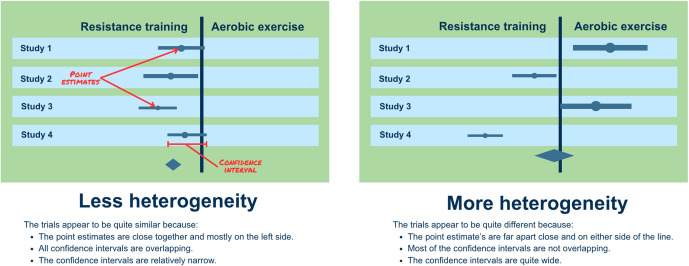
Visually inspecting a forest plot.

These concepts are explained further in Fig 3.

After visually inspecting the forest plot, we will also measure the heterogeneity using a calculation called the I^2^ estimate, which is represented as a percentage (%). We will use the thresholds outlined in Table 3 [69]. There is overlap across the thresholds, as I^2^ estimates should be interpreted cautiously based on the context.

**Table 3:** I^2^ estimate thresholds.

Threshold	Meaning
75-100%	Considerable heterogeneity – meaning the studies may be very different from each other
50-90%	Substantial heterogeneity – meaning the studies may be somewhat different from each other.
30-60%	Moderate heterogeneity – meaning there is some difference that may be important.
0-40%	The studies are fairly similar, and the amount of difference is possibly not relevant.

We will also complete a statistical test called Tau-squared. Tau-squared measures variance between studies in a meta-analysis. Tau-squared is presented as a number with larger values indicating more variation between studies, and therefore more heterogeneity [69].

Finally, we will complete another statistical calculation called the chi-squared test. The chi-squared test can assess whether the difference between the studies is due to chance alone or for other reasons. It is presented as a number called a p-value. The lower the p-value (the closer to zero), the higher the probability that the heterogeneity is likely not due to chance and is statistically significant [69].

We will summarise our assessment of heterogeneity in a written summary, including possible causes of heterogeneity. If we observe high levels of heterogeneity, indicating that the studies are too dissimilar to pool together, we will not complete a meta-analysis. Performing a meta-analysis of trials that are too different would not be appropriate and may misrepresent the study’s results. We will report the results in this case in written and table format, following best practice guidelines [65].

### How will we check if trials are missing? - assessment of reporting biases

A common problem with research is that sometimes trials with less favourable results are not published, and the results are hidden from the public. This is known as reporting bias. Reporting bias can impact the results of a systematic review, as it might seem like one treatment (the one with more published trials) is more effective than it actually is.

**Fig 4:**
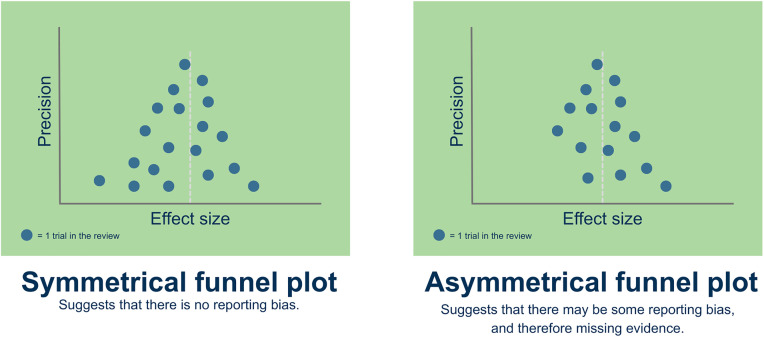
How to interpret a funnel plot.

To check for reporting bias, we will follow the guidance in the Cochrane Handbook [70]. We will use a funnel plot if there are more than 10 studies included in our meta-analysis [71,72]. A funnel plot (Fig 4) can be used to explore whether reporting bias is present. If the funnel plot’s shape is symmetrical, then this indicates an absence of reporting bias. If it is asymmetrical, then this suggests that results are grouped, telling “one side” of the story, or showing only the positive findings – there may be some reporting bias or hidden results, and, therefore, missing evidence. Besides analysing the plot visually, we will check for funnel plot asymmetry using a statistical test called an Egger test [73]. The *Technical Team* will prepare the funnel plot and complete the Egger test, and *the People* will help interpret the funnel plot. If there are fewer than 10 studies available funnel plots are unreliable, so we will search trial registries for unpublished studies. We will also compare published study reports with pre-determined plans (for example trial registry information, protocols or statistical analysis plans).

### How will we combine the results from each study together? - data synthesis

If the studies are sufficiently similar, they will be combined using meta-analysis. We will perform a separate meta-analysis for each outcome – quality of life and heart health (measured by functional fitness). We will use a website called MetaAnalysisOnline.com [74] to conduct the meta-analysis as it is freely available and open access.

One member of the *Technical Team* will enter the data into MetaAnalysisOnline.com and another will check that the data was inputted accurately. The meta-analysis will then be conducted in two steps.

- Step 1: Calculate the measure of effect for each study using either the mean difference or standardised mean difference (as described in the section above: *Measuring the effects of the intervention versus the comparator*).
- Step 2: Combine the effect measures using the random-effects model using the inverse-variance method. There are different types of statistical models to use for a meta-analysis – we will use the random-effects model because it takes into consideration any difference (heterogeneity) across the studies.

If meta-analysis is not appropriate, for example, if there are lots of missing data, or if the studies are very different from each other, then we will describe the results through written word, tables, and graphs. We will follow best practice guidelines for explaining the results in this way – this guidance is known as SWiM (Synthesis without meta-analysis; [65]. The *Technical Team* will conduct the synthesis (either by meta-analysis or following SWiM guidelines) and *the People* will help interpret it.

### Will we look at groups of studies with specific aspects? - subgroup analysis

As decided by *the People*, we will conduct a sub-group analysis to explore differences:

- Across sex and/or genders (based on what is most commonly reported in the trials).
- In age categories between 50–60 years and 60 + years.
- Between healthy participants and participants with reported health conditions.

The *Technical Team* will only conduct a sub-group analysis if there are sufficient data (from at least 2 studies) available in each sub-group [75] and will be considered exploratory.

### How will we explore whether studies with high risk of bias made a difference to the results? - sensitivity analysis

A sensitivity analysis will explore the influence of studies with a high risk of bias in any domain. We will achieve this by excluding studies with a high risk of bias from the analysis and examining the differences in the overall results. This will be done by the *Technical Team* using MetaAnalysisOnline.com [74], and it will be reported in a table, or forest plot. If cluster randomised trials are included, we will also conduct a sensitivity analysis excluding the studies that did not account for clustering in their analysis.

### How will we determine how sure we can be that resistance training makes a difference to the quality of life or heart health for older adults compared to aerobic exercise? - assessment of the certainty of evidence

In a systematic review, we determine how confident we can be in the results or how sure we are that the evidence gathered by the review is a good representation of the likely effect of the intervention (resistance training). This is called certainty of evidence. [76].

To assess the certainty of evidence for our review, we will use the GRADE (Grading of Recommendations, Assessment, Development and Evaluation) approach [77]. GRADE uses a transparent and organised approach to rating the certainty of evidence in four categories: high, moderate, low and very low certainty of evidence.

For the GRADE approach, if all the studies are randomised trials, then we start with high certainty of evidence. However, the rating can decrease if there are issues. GRADE looks at five domains which may lead to reducing the certainty of evidence. These domains are described in Table 4.

**Table 4:** Domains that can rate down the certainty of evidence in the GRADE approach.

Domain	What it is	Why it matters	How it is assessed
**Risk of bias**	There are flaws in how the studies were done.	If the studies were not well done and there is a high risk of bias, the results may be less reliable [78].	Using the risk of bias assessment described in section: *How will we check for bias in the studies? – risk of bias*
**Imprecision**	When the studies have very small numbers of participants, or the confidence intervals are very wide.	If the results are too imprecise we cannot be confident in the size of the effect [79].	Checking the width of the confidence interval if the effect size (usually the diamond in the forest plot) and by asking ourselves do we have enough participants overall to be confidence in the results?
**Indirectness**	When the studies don’t exactly match the question of interest (e.g., different population, interventions or outcomes).	If the evidence doesn’t directly address the question we’re asking, it is not very useful for answering it [80].	By looking at the population, interventions and outcomes in the studies and asking ourselves do these match the review question?
**Inconsistency**	When the studies give very different results.	If the studies give very different results it is much harder to know whether the intervention actually works or not [81].	By checking if studies show similar results in both direction and size, and by using statistical tests (like I²) to measure how much they differ.
**Publication bias**	When there are studies missing (usually those showing no effect or negative results)	If only certain results are available, the evidence may only be telling one side of the story and show misleading results [78].	Using a funnel plot (and statistical tests for asymmetry) as described in the section: *Assessment of reporting bias*).

Both outcomes, quality of life and heart health (measured by functional fitness), will be assessed using this GRADE approach by the *Technical Team* and presented in a “summary of findings” table. Two people will rate the certainty of evidence for each outcome separately, and if they have different opinions, they will discuss until they agree. If agreeing on the GRADE rating is too difficult, they will consult with a third member of the team to decide.

GRADE results of a systematic review are usually presented in a table called “summary of findings”, to make it easier for the user to understand the overall review results quickly. The information to be included in the summary of findings tables for each outcome includes:

- The number of trials and the number of participants included in the analysis.
- The estimated effect (measured using either the mean difference or the standardised mean difference) and 95% confidence interval. The effect estimate will be reported to either have or not have an effect in line with best practice guidelines [82]. This is known as a threshold of “no effect”.
- The overall GRADE certainty of evidence rating (high, moderate, low or very low) with an explanation for this rating.
- Plain language summary explaining what the GRADE rating means and contextualising this to everyday health decision making if possible.

The GRADEpro GDT software [83] will be used to create the summary of findings tables. If it is not possible to complete a meta-analysis we will present a narrative description of the effects in the summary of findings table and rate the certainty of evidence using alternative methods [84]. The *Technical Team* will lead the assessment of certainty with input from *the People* in interpreting the five domains highlighted in Table 4.

### How will we consider equity in our review?

We know that physical activity and exercise are beneficial for older adults’ physical and mental health. However, these benefits may not be equally experienced by everyone, and we must explore factors that could make accessing exercise more challenging for some people. For example, the number of people who engage in enough exercise varies based on specific factors such as sex/gender, socioeconomic status, and race/ethnicity [85–87]. These factors may be considered as health inequities, as they are inequalities between groups that are both unfair and avoidable [88].

Studies to date, exploring the effects of physical activity among older adults, have seldom explored how the differences in the effects of these interventions are based on factors related to health inequity [89,90]. Therefore, within this systematic review (and systematic reviews in general), it is important to accurately and consistently present equity-related data so that users of our review can interpret the results within their specific context.

For this review, we will use the PRO-EDI [91–93] tool and guidance from the Cochrane Handbook [94] to inform our consideration of health equity. Specifically, we will:

- Extract information from the included trials related to participant characteristics that may contribute to inequitable access to the intervention.
- Report this information in a participant characteristics table [92], including information about the people we would expect to see in the studies, and data reflecting the people who took part in the studies.
- Include information in the summary of findings table related to equity, outlining the populations to whom the certainty of evidence assessment applies (and may not apply).
- Conduct several subgroup analyses related to factors that are known to contribute to inequitable access to exercise interventions for older adults, including sex/gender, disability/presence of health conditions, and location (low, middle and high income countries).
- Interpret the findings and applicability of the review results related to health equity in the discussion section.

### How will we share data and results from our review?

We will write-up and share the results of this review in plain English and submit it to a peer-reviewed, open-access journal so that the review is accessible to as many people as possible. We will share a pre-print of the review before it is submitted to the journal, so the results are available as soon as possible. We will co-create with *the People* material to share the results in accessible formats on our website, through social media and other channels. Additionally, we will share the data generated from this review freely and openly in OSF including a permanent shareable link to the meta-analysis conducted within MetaAnalysisOnline.com [74].

## Discussion

This review is being produced as an output from The People’s Review project [1]. We propose here the methods for a systematic review conducted by the public about a topic chosen by the public, investigating whether resistance training makes a difference to quality of life or heart health (measured using functional fitness) compared to aerobic exercise, for older adults (over the age of 50 years). To the best of our knowledge, no high-quality previous systematic review has explored this topic with such level of public participation in planning and conducting the review. We hope that this review will be of use to people making decisions about engaging in different types of exercise in their later years, including older adults themselves, healthcare professionals, researchers, policymakers, and funders.

There is growing support for members of the public to be involved in health research, including systematic reviews [95–98], with a positive impact for everyone involved, including members of the public, researchers, and healthcare professionals alike [95,99–101]. We aim to produce a high-quality review, co-created with the public, and support the public’s understanding of systematic reviews and their importance. We hope that our review will encourage other review authors to consider including patient and public members in the review process and consider using The People’s Review method as a new way of involving people in health research. We will report the findings from The People’s Review processes in future publications.

This protocol has several strengths, including that it follows best practice guidelines for systematic reviews about healthcare interventions [102,103] and that it actively involves the public throughout the whole review process. However, as with all research, this review may have some limitations. One scientific limitation of this review is that there are many ways to measure heart health, and functional fitness is only one (albeit important) measure of heart health. However, this review is being led by *the People*, who considered this as the most important outcome. Future reviews may consider exploring other clinically important heart health measures, including blood pressure changes, changes in structural heart health, cholesterol level or other measures of fitness. A limitation of this review that may be considered is that we are not using the most up-to-date version of the Risk of Bias tool – ROB-2 [59]. ROB-2 has been criticised as being overly complicated, requiring extensive training, and there has been limited implementation of the tool among systematic reviewers [60–63]. Therefore, for this review we will use the original Risk of Bias tool [58] as it is similar to the updated ROB-2 as it assesses the same domains, but is structured differently and in a more accessible and easier to use way – especially for people without experience. We will address one of the main limitations of the original tool by assessing each outcome (quality of life and heart health) instead of assessing the risk of bias for each study as a whole. Future reviews could consider using the updated tool [59] or ROBUST-RCT [104] another recently published tool for assessing risk of bias in randomised trials.

## Supporting information

S1 File Search Strategy. (DOCX)

S2 File PRISMA-P Checklist. (DOCX)
