# Supplementary material for: Does resistance training make a difference to the quality of life or heart health for older adults compared to aerobic exercise? A systematic review protocol from The People’s Review
Source: PLoS One. 2026 Jun 10;21(6):e0337017. doi: 10.1371/journal.pone.0337017 (PMC13252789; doi:10.1371/journal.pone.0337017)
Supplement: S1 File — (DOCX) [file pone.0337017.s001.docx]

**Supplementary Material 1. Search Strategy**

Database name: **Medline**

Search performing date: 08/10/2025

Interface: Ovid

Database timeframe: 1946 to 29/09/2025

Search screen: Advanced screen

Search modes: Boolean / Phrase / Proximity

Filters used:

- Cochrane sensitivity and precision maximising search filter for randomised controlled trials - Glanville J, Kotas E, Featherstone R, Dooley G. Which are the most sensitive search filters to identify randomized controlled trials in MEDLINE? J Med Libr Assoc 2020;108:556–63. https://doi.org/10.5195/jmla.2020.912.
- Wright and Jones search filter for older adults (over 50 years of age) - Wright JM, Jones D. Search strategies to investigate decision making by older adults and appraisal by GPs following cancer symptoms 2021. <https://doi.org/10.5518/1034>.

| **No #** | **Search** | **Results** |
| --- | --- | --- |
| 1 | resistance training/ | 14739 |
| 2 | weight lifting/ | 5367 |
| 3 | ((weight$ or power$) adj2 lift$).tw. | 2350 |
| 4 | (weightlift$ or powerlift$).tw. | 1706 |
| 5 | ((resistance or resistive or strength$ or weight$ or bodyweight or body weight or anaerob$) adj2 (coach$ or train$ or exercis$ or conditioning or workout$ or program$)).tw. | 52691 |
| 6 | (progressive adj2 (overload$ or resistance)).tw. | 1880 |
| 7 | (calisthenic$ or callisthenic$).tw. | 330 |
| 8 | 1 or 2 or 3 or 4 or 5 or 6 or 7 | 60321 |
| 9 | endurance training/ | 793 |
| 10 | cardiorespiratory fitness/ | 4105 |
| 11 | high-intensity interval training/ | 2914 |
| 12 | ((aerob$ or cardio$ or respirat$ or endur$) adj2 (coach$ or train$ or exercis$ or activit$ or workout$ or fitness or program$)).tw. | 80687 |
| 13 | (HIIT or HIIE).tw. | 3414 |
| 14 | ((high or low or medium or moderate) adj2 intens$ adj2 (train$ or exercis$ or activit$ or workout$)).tw. | 20963 |
| 15 | ((gait or walk$ or treadmill$ or BWSTT or ambulat$ or cycl$ or ergomet$ or run$ or jog$ or elliptic$ or bike or bicycl$ or cycl$ or swim$) adj2 (coach$ or train$ or exercis$ or activit$ or workout$ or program$ or fitness$)).tw. | 81567 |
| 16 | 9 or 10 or 11 or 12 or 13 or 14 or 15 | 167999 |
| 17 | exp Aged/ | 3764745 |
| 18 | elderly.tw,kw. | 325524 |
| 19 | geriatric*.tw,kw. | 88747 |
| 20 | senior.tw,kw. | 48995 |
| 21 | (older adj (adult? or m#n or wom#n or person? or people)).tw,kw. | 225715 |
| 22 | Geriatrics/ | 32130 |
| 23 | (Age? adj3 (over or older) adj2 (5# or 6# or 7# or 8# or 9#)).tw. | 63806 |
| 24 | sexagenarian.tw,kw. | 82 |
| 25 | septuagenarian.tw,kw. | 277 |
| 26 | octogenarian.tw,kw. | 2045 |
| 27 | nonagenarian.tw,kw. | 843 |
| 28 | centenarian.tw,kw. | 1021 |
| 29 | gerontolog*.tw,kw. | 9950 |
| 30 | (">=5# years old" or ">5# years old").tw. | 20016 |
| 31 | (">=6# years old" or ">6# years old").tw. | 32083 |
| 32 | (">=7# years old" or ">7# years old").tw. | 18812 |
| 33 | (">=8# years old" or ">8# years old").tw. | 11537 |
| 34 | (">=9# years old" or ">9# years old").tw. | 3830 |
| 35 | 17 or 18 or 19 or 20 or 21 or 22 or 23 or 24 or 25 or 26 or 27 or 28 or 29 or 30 or 31 or 32 or 33 or 34 | 4021905 |
| 36 | exp randomized controlled trial/ | 649290 |
| 37 | controlled clinical trial.pt. | 95742 |
| 38 | randomized.ab. | 712020 |
| 39 | placebo.ab. | 262627 |
| 40 | clinical trials as topic.sh. | 205896 |
| 41 | randomly.ab. | 470482 |
| 42 | trial.ti. | 347605 |
| 43 | 36 or 37 or 38 or 39 or 40 or 41 or 42 | 1718986 |
| 44 | exp animals/ not humans.sh. | 5381851 |
| 45 | 43 not 44 | 1585871 |
| 46 | 8 and 16 and 35 and 45 | 1696 |
