# Supplementary material for: Does resistance training make a difference to the quality of life or heart health for older adults compared to aerobic exercise? A systematic review protocol from The People’s Review
Source: PLoS One. 2026 Jun 10;21(6):e0337017. doi: 10.1371/journal.pone.0337017 (PMC13252789; doi:10.1371/journal.pone.0337017)
Supplement: Attachment — Submitted filename: Response_to_Reviewers.pdf [file pone.0337017.s004.pdf]

Ollscoil na Gaillimhe
University of Galway

Dear Editor,

Many thanks for reviewing this manuscript - PONE-D-25-58754 - *Does resistance training make a difference to the quality of life or heart health for older adults compared to aerobic exercise? A systematic review protocol from The People's Review*. We are grateful for the time spent from the editorial office and peer-reviewers reviewing and sharing feedback on this manuscript. Please find below a line-by-line response to the editor and peer-reviewer's comments. We have updated the manuscript where appropriate also. Thank you for considering this manuscript.

All the best,

Éle Quinn [1,2,3] - on behalf of the authors of this manuscript.
Email: e.quinn34@universityofgalway.ie –

[1]Discipline of Occupational Therapy, School of Health Sciences, University of Galway, Galway, Ireland.
[2]Evidence Synthesis Ireland and Cochrane Ireland, University of Galway, Galway, Ireland.
[3]Centre for Health Research Methodology, School of Nursing and Midwifery, University of Galway, Galway, Ireland.

Peer-reviewer major comments	Author response
1. Conceptual Framing of "Heart Health"<br>You define heart health exclusively as functional fitness (e.g., 6MWT).<br>Strengths<br>• Clear justification based on public prioritisation.<br>• Transparent acknowledgement that other cardiac outcomes exist.<br>Concerns<br>• Functional fitness is not a direct measure of cardiac physiology.<br>• It may reflect musculoskeletal, pulmonary, motivational, and neurological contributions.<br>• The conceptual shift from "heart health" to "functional aerobic capacity" could be misinterpreted.<br>Recommendation<br>Strengthen internal consistency by:<br>• Explicitly stating that heart health is operationalised as functional fitness for this review.<br>• Adding one clarifying sentence noting that functional fitness is a proxy measure rather than a direct cardiac biomarker.<br>This will protect against criticism from cardiology-focused reviewers.	Thank you for this helpful comment. We agree that functional fitness is not a direct physiological measure of cardiac function. To improve clarity of this and to reduce the risk of misinterpretation, we have revised parts of the Outcomes section.<br><br>See lines 242-264:<br>"There are many outcomes we could use to measure heart health – blood pressure, resting heart rate, fat (or lipid) markers such as cholesterol, VO_2max (the maximum amount of oxygen the body can use during exercise) and many more. However, for this review we will use functional fitness as the measure for heart health. The People prioritised several heart health outcomes and selected functional fitness as the outcome to focus on. Functional fitness (or functional aerobic capacity) is a person's ability to keep exercising over a sustained time period, at a level where they can still talk without becoming very breathless. It is one of the best measures of overall fitness and heart health as it evaluates all the body's systems working together during exercise (49,50). While functional fitness does not directly measure heart function through biological markers like blood pressure and cholesterol levels, it is a valuable measure of overall heart health. We will include studies that measure functional fitness through accepted tests such as the 6-minute walk test (6MWT), shuttle tests or other methods.<br><br>No other outcomes will be explored in this review. We acknowledge that this review could focus on many other outcomes related to heart health. For this review we are focusing on functional fitness only as the measure of heart health. The People decided to focus only on functional fitness, as this was considered the most important measure of heart health for them."
2. Restriction to ≥8 Sessions<br>The eligibility criterion of ≥8 sessions is clearly justified as public-chosen.<br>However:<br>• Why 8 sessions specifically?<br>• Is there empirical precedent?<br>• Could this exclude short but physiologically meaningful RCTs?<br>Recommendation	Thank you for raising this point. We have now added additional justification for the ≥8-session threshold in the eligibility criteria. This threshold was selected the public to and reflects a balance between meaningful exercise dose while remaining feasible in most settings. The manuscript has been revised for clarification. See lines 227-232.<br><br>"We will include trials that compare resistance training with aerobic exercise where the programme includes at least

Add one brief methodological justification such as:<br>• Eight sessions approximates a minimal exposure threshold for physiological adaptation.<br>• Or clarify that this threshold balances feasibility and meaningful intervention dose.<br>Without rationale, this may appear arbitrary.	eight sessions, regardless of how often (frequency) and how long (duration) each session was. The People chose this threshold because it provides enough time for participants to receive enough exercise for their bodies to adapt and show changes in fitness, while still being feasible to carry out in real-world settings. Trials with fewer than eight sessions will be excluded from this review."
3. Risk of Bias Tool (ROB-1 vs ROB-2)<br>Your justification for using ROB-1 is thoughtful and transparent.<br>However:<br>• ROB-1 uses "unclear risk"<br>• Your manuscript uses "some concerns" (ROB-2 terminology)<br>This may create conceptual inconsistency.<br>Recommendation<br>Ensure terminology matches ROB-1 exactly:<br>• Low risk<br>• High risk<br>• Unclear risk<br>Or clearly state if modified terminology is used.<br>Also consider adding:<br>"The domains assessed align conceptually with ROB-2, though structured differently."<br>This will reassure methodologists.	Thank you for highlighting this inconsistency. We have now amended the terminology throughout the manuscript to align fully with the ROB-1 tool.<br><br>See line 381:<br>"For each domain, we will assign a judgement of either low, high or unclear risk of bias."<br><br>Additionally, we have added in additional information in the Limitations section to highlight that the ROB-2 is conceptually similar to ROB though structured differently.<br><br>See lines 672-682:<br>"A limitation of this review that may be considered is that we are not using the most up-to-date version of the Risk of Bias tool – ROB-2 (59). ROB-2 has been criticised as being overly complicated, requiring extensive training, and there has been limited implementation of the tool among systematic reviewers (60–63). Therefore, for this review we will use the original Risk of Bias tool (58) as it is similar to the updated ROB-2 as it assesses the same domains, but is structured differently and in a more accessible and easier to use way - especially for people without experience. We will address one of the main limitations of the original tool by assessing each outcome (quality of life and heart health) instead of assessing the risk of bias for each study as a whole. Future reviews could consider using the updated tool (59) or ROBUST-RCT (104) another recently published tool for assessing risk of bias in randomised trials."
4. Citizen Science Model – Methodological Safeguards<br>This is the most innovative aspect of your review.<br>You describe:<br>• Training modules<br>• 4-person extraction<br>• 80% agreement threshold	Thank you for this helpful feedback on the citizen science model. As suggested, we have revised the manuscript to clarify both the agreement algorithm and the training procedures used to support the public.<br><br>We specify that, for data extraction, the 80% agreement threshold is calculated per data item. If fewer than 80% of contributors extract the same value for a given item, the

• Technical resolver<br>This is strong.<br>However, two clarifications would strengthen rigor:<br>A. Agreement Algorithm<br>How is 80% calculated?<br>• Per data item?<br>• Per study?<br>• Per domain?<br>Clarify the unit of agreement.<br>B. Bias Risk in Public Assessment<br>Public contributors assess first 4 ROB domains.<br>Potential issues:<br>• Understanding allocation concealment<br>• Understanding sequence generation<br>You might briefly clarify:<br>• Whether calibration exercises will be conducted.<br>• Whether example worked cases are provided.<br>These additions would enhance credibility.	discrepancy is flagged and resolved by a member of the Technical Team. See lines 348-354 for details.<br><br>"People may extract slightly different information from each study. When there is less than 80% agreement for each data item extracted by the People, a resolver from the Technical Team will make the final decision. If the Technical Team members do not agree, this disagreement will be resolved through discussion or involving a third team member if necessary."<br><br>For the risk of bias assessment, the 80% agreement threshold is applied per domain. Each study will be assessed independently by at least four public contributors. Where agreement falls below the 80% threshold, the Technical Team will review the judgement and make the final decision. See lines 387-392.<br><br>"The People will look at the first four risk of bias domains, with the remaining three domains to be assessed independently by two members of the Technical Team. Each study will be assessed by at least four different members of the public. If there is less than 80% agreement for each domain assessed by the People, a member of the Technical Team will act as a resolver to make the final decision. If discrepancies arise in the Technical Team, they will resolve it through discussion or with a third assessor."<br><br>We have also expanded the description of training and calibration procedures to clarify that public contributors will complete structured training modules, worked examples, and practice exercises before undertaking risk-of-bias assessments (lines 381-385).<br><br>"For each domain, we will assign a judgement of either low, high or unclear risk of bias. As with previous stages, bespoke training will be available to explain key concepts about risk of bias, provide worked examples and allow people to practice before assessing the risk of bias in the included studies. The public can assess the risk of bias in as many studies as they choose; however, they will be asked to assess the risk of bias in at least one study."
5. Meta-Analysis and SMD Interpretation<br>You appropriately avoid Cohen's	Thank you for raising this point. In line with recent GRADE guidance (Hultcrantz et al., 2025), we will interpret effect

small/medium/large thresholds.<br>This aligns with contemporary GRADE thinking.<br>However, for readers:<br>• If SMD is 0.25 or 0.40, how will this be interpreted narratively?<br>Recommendation:<br>Add one sentence clarifying that:<br>Effect sizes will be interpreted relative to the threshold of no effect and contextualised clinically where possible.<br>This prevents the impression that magnitude interpretation is avoided entirely.	estimates relative to the threshold of no effect rather than using minimally important difference. To clarify this approach, we have added additional text explaining how effect estimates and confidence intervals will be interpreted using GRADE (lines 588–600).<br><br>"GRADE results of a systematic review are usually presented in a table called "summary of findings", to make it easier for the user to understand the overall review results quickly. The information to be included in the summary of findings tables for each outcome includes:<br><br>• The number of trials and the number of participants included in the analysis.<br>• The estimated effect (measured using either the mean difference or the standardised mean difference) and 95% confidence interval. The effect estimate will be reported to either have or not have an effect in line with best practice guidelines (82). This is known as a threshold of "no effect".<br>• The overall GRADE certainty of evidence rating (high, moderate, low or very low) with an explanation for this rating.<br>• Plain language summary explaining what the GRADE rating means and contextualising this to everyday health decision making if possible."
6. Handling of Cluster and Crossover Trials<br>Your approach is appropriate.<br>However, clarify:<br>• For cluster trials: will ICC be extracted or assumed?<br>• If ICC not reported, will external ICC estimates be used?<br>A single clarifying sentence would strengthen analytic transparency.	Thank you for this useful suggestion. We have added further detail to Table 2 describing how cluster-randomised trials will be handled in this review – specifically addressing the intracluster correlation coefficient (ICC).<br><br>See *Table 2. Study designs that need to be accounted in this review* for details:<br><br>"The Technical Team will assess if the authors of the cluster trials have appropriately accounted for clustering and get advice from a statistician if needed. If the authors have not appropriately accounted for clusters, we will adjust for this using methods recommended in the Cochrane handbook. These methods require specific data (such as the intracluster correlation coefficient (ICC)) to make appropriate adjustments. If this is not reported in the study we will contact the study authors or use external estimates and clearly document the source used (66)."

7. Heterogeneity Interpretation<br>The I^2 table is clearly presented.<br>However:<br>• Overlap in thresholds is acknowledged, which is good.<br>• Consider adding Tau^2 (between-study variance) as a secondary descriptor.<br>Even if not emphasised publicly, noting it strengthens statistical rigor.	Thank you for this suggestion. We have now added Tau^2 (between study variance) as an additional measure of heterogeneity. See lines 493-496 for details.<br><br>"We will also complete a statistical test called Tau-squared. Tau-squared measures variance between studies in a meta-analysis. Tau-squared is presented as a number with larger values indicating more variation between studies, and therefore more heterogeneity (69)."
8. Reporting Bias and Funnel Plot<br>Appropriate threshold of >10 studies.<br>Clarify:<br>• If fewer than 10 studies, reporting bias will be discussed qualitatively.<br>This prevents methodological gaps.	Thank you for highlighting this point. We have added text clarifying that when fewer than 10 studies are available. See lines 518-521.<br><br>"If there are fewer than 10 studies available funnel plots are unreliable, so we will search trial registries for unpublished studies. We will also compare published study reports with pre-determined plans (for example trial registry information, protocols or statistical analysis plans)."
9. Data Availability Statement<br>You now include OSF sharing and permanent links.<br>Strong transparency.<br>One suggestion:<br>Specify whether:<br>• Extracted dataset will be shared<br>• Risk of bias assessments will be shared<br>• Agreement metrics will be shared<br>That would elevate open science quality.	We have now included additional information in the data availability statement to clarify that extracted data, risk of bias assessments and results from agreement metrics will be published once the review is complete. See data availability statement below:<br><br>"Data collected during this review process including extracted data, risk of bias assessment and decisions from agreement algorithms, will be publicly available when the review is completed and published."

**Peer-reviewer minor comments**	**Author response**
1. Terminology Consistency<br>Occasional variation between:<br>"Older adults"<br>"People over 50"<br>"Adults over 50"<br>Choose one primary term and maintain consistency.	Thank you for flagging this. We have now amended the manuscript to use the term older adults consistently throughout and explained in the eligibility criteria that for this review we use the threshold of over 50 as our definition of older adult.
2. Quality of Life Instruments<br>You mention SF-36, EuroQol, WHOQOL.<br>Consider clarifying:<br>• Whether mental and physical component	Thank you for highlighting this point. We have clarified that where quality-of-life instruments report separate mental and physical component scores, these domains will be

Ollscoil na Gaillimhe
University of Galway

scores will be analysed separately if reported.	extracted and analysed separately where appropriate. See lines 244-246 for details.<br>"These measures often include different areas including both the mental and physical aspects of quality of life. Both mental and physical health aspects will be collected and analysed separately, if available."
3. Gender vs Sex Data Extraction<br>You list:<br>• sex<br>• gender<br>• sexual identity<br>Strong equity inclusion.<br>Clarify whether:<br>• Analyses will distinguish sex vs gender where reported.	Thank you for raising this. Sex and gender will be distinguished where reported, and this information will be presented in a participant characteristics table in line with PRO-EDI guidance. This is outlined in the data extraction and health equity sections.
4. Language<br>• The plain English style is excellent and consistent.<br>• However, a few places could be slightly tightened for conciseness without losing accessibility.<br>• For example:<br>• "We are all one large review team…"<br>• Could be shortened slightly to improve flow.	We appreciate this suggestion. The manuscript has been reviewed and revised to improve clarity and conciseness where possible while maintaining accessibility.
5. Figures<br>Ensure that:<br>• Fig. 1 flow diagram<br>• Fig. 2 forest plot explanation<br>• Fig. 3 heterogeneity explanation<br>Are formatted according to PLOS ONE graphical standards.	All figures have now been reviewed and formatted in accordance with PLOS ONE graphical guidelines.
