# Supplementary material for: Does resistance training make a difference to the quality of life or heart health for older adults compared to aerobic exercise? A systematic review protocol from The People’s Review
Source: PLoS One. 2026 Jun 10;21(6):e0337017. doi: 10.1371/journal.pone.0337017 (PMC13252789; doi:10.1371/journal.pone.0337017)
Supplement: Attachment — Submitted filename: Response to Reviewers.docx [file pone.0337017.s003.docx]

­

Dear Editor,

Many thanks for reviewing this manuscript - PONE-D-25-58754 - *Does resistance training make a difference to the quality of life or heart health for older adults compared to aerobic exercise? A systematic review protocol from The People's Review*. We are grateful for the time spent from the editorial office and peer-reviewers reviewing and sharing feedback on this manuscript. Please find below a line-by-line response to the editor and peer-reviewer's comments. We have updated the manuscript where appropriate also. Thank you for considering this manuscript.

All the best,

Éle Quinn ^1,2,3^ - on behalf of the authors of this manuscript.

Email: [e.quinn34@universityofgalway.ie](mailto:e.quinn34@universityofgalway.ie) –

^1^Discipline of Occupational Therapy, School of Health Sciences, University of Galway, Galway, Ireland.

^2^Evidence Synthesis Ireland and Cochrane Ireland, University of Galway, Galway, Ireland.

^3^Centre for Health Research Methodology, School of Nursing and Midwifery, University of Galway, Galway, Ireland.

**Table 1. Line-by-line response**

| **Journal Requirement** | **Author Response** |
| --- | --- |
| 1. Please ensure that your manuscript meets PLOS ONE's style requirements, including those for file naming. The PLOS ONE style templates can be found at  <https://journals.plos.org/plosone/s/file?id=wjVg/PLOSOne_formatting_sample_main_body.pdf> and  [https://journals.plos.org/plosone/s/file?id=ba62/PLOSOne_formatting_sample_title_authors_affiliations.pdf](https://track.editorialmanager.com/CL0/https:%2F%2Fjournals.plos.org%2Fplosone%2Fs%2Ffile%3Fid=ba62%2FPLOSOne_formatting_sample_title_authors_affiliations.pdf/1/010f019b9baac6d4-5837c0a1-9810-4c92-ba0b-b69782cf9d23-000000/XpaguAj0jt1DMbPk-Wt8tvEhBrcLhPTSI6gkzb-_2Q8=245) | The manuscript has now been revised in line with PLOS ONE’s style requirements. |
| 2. We note that the grant information you provided in the ‘Funding Information’ and ‘Financial Disclosure’ sections do not match.  When you resubmit, please ensure that you provide the correct grant numbers for the awards you received for your study in the ‘Funding Information’ section. | The Funding Information has now been updated to match the Financial Disclosure within the manuscript (lines 684-690). The following funders have been added to the Funding Information: the HSC Research and Development Division of the Public Health Agency (Northern Ireland), College of Medicine, Nursing and Health Sciences, University of Galway (Ireland) and Evidence Synthesis Ireland. No grant number is available for funding from these agencies. |
| 3. One of the noted authors is a group or consortium “The People”. In addition to naming the author group, please list the individual authors and affiliations within this group in the acknowledgments section of your manuscript. Please also indicate clearly a lead author for this group along with a contact email address. | Thank you for raising this point. The lead author of this group is Ele Quinn and if someone wishes to contact the group they can email: [thepeoplesreview@universityofgalway.ie.](mailto:thepeoplesreview@universityofgalway.ie)  In line with our ethics approval (2023.06.012), we did not collect individual affiliation data for members of this group, and we do not have consent to publicly disclose this information. Many contributors are members of the public and do not hold academic or institutional affiliations. For these reasons, we are unable to list the affiliations for the group in the Acknowledgments section. The individual authors names are listed in the Acknowledgments section. As per the journal requirements <https://journals.plos.org/plosone/s/file?id=ba62/PLOSOne_formatting_sample_title_authors_affiliations.pdf> it is not required for group authors affiliations to be listed. |
| 4. Your ethics statement should only appear in the Methods section of your manuscript. If your ethics statement is written in any section besides the Methods, please move it to the Methods section and delete it from any other section. Please ensure that your ethics statement is included in your manuscript, as the ethics statement entered into the online submission form will not be published alongside your manuscript. | Many thanks for flagging this. The ethics statement is now included in the Methods section from line 169-171. |
| 5. If the reviewer comments include a recommendation to cite specific previously published works, please review and evaluate these publications to determine whether they are relevant and should be cited. There is no requirement to cite these works unless the editor has indicated otherwise. | Thank you for this comment. Reviewer comments do not include a recommendation to cite specific previously published works. |

| **Reviewer #1 Comments** | **Authors Response** |
| --- | --- |
| This is a well-constructed, clearly written systematic review protocol with an innovative and commendable emphasis on citizen science and public involvement throughout all stages of the review. The plain-language style improves accessibility and transparency. The methodology largely aligns with Cochrane standards and PRISMA-P guidance, with appropriate attention to bias, heterogeneity, equity, and certainty of evidence. Below are major and minor comments intended to strengthen clarity, rigor, and reproducibility. | Many thanks for your considerate and useful comments. We are especially grateful for your recognition of the citizen science approach, the emphasis on public involvement, and the use of plain-language reporting to enhance accessibility and transparency.    We have carefully considered all major and minor comments and have revised the manuscript accordingly to strengthen clarity, methodological rigor, and reproducibility. Point-by-point responses to each comment are provided below, with corresponding changes tracked in the revised manuscript. |
| **Major Comments** | |
| 1. Scope and rationale — very well justified, but clarify outcome definitions  You clearly explain why comparing resistance vs aerobic training in adults ≥50 years matters. However, the decision to use functional fitness as the sole measure of “heart health” deserves further clarification:  • Functional fitness reflects overall capacity rather than cardiac function alone  • Other cardiac outcomes (e.g., BP, VO₂max, LV function, lipid markers) are clinically meaningful  Suggestion: Add a brief justification explaining why functional fitness was prioritised over physiologic cardiac endpoints (e.g., relevance to the public, feasibility, comparability). | Thank you for this helpful comment. We agree that heart health can be assessed in many ways. As suggested, we have updated the manuscript to provide additional clarification and context for the decision to use functional fitness as the sole measure of heart health in this review.    Additional information provided in lines 244-257.    There are many outcomes we could use to measure heart health – blood pressure, resting heart rate, fat (or lipid) markers such as cholesterol, VO₂max (the maximum amount of oxygen the body can use during exercise) and many more. However, for this review we will use functional fitness as the measure for heart health. The People prioritised several heart health outcomes and selected functional fitness as the outcome to focus on. Functional fitness (or functional aerobic capacity) is a person’s ability to keep exercising over a sustained time period, at a level where they can still talk without becoming very breathless. It is one of the best measures of overall fitness and heart health as it evaluates all the body’s systems working together during exercise (49,50). We will include studies that measure functional fitness through accepted tests such as the 6-minute walk test (6MWT), shuttle tests or other methods. |
| 2. Inclusion criteria — clarify mixed-age samples  You state trials including participants <50 years will be included only if over-50 data are reported separately. Many trials do not stratify by age.  Suggestion: State explicitly how such studies will be handled if stratified data are unavailable, whether authors will be contacted, and whether these trials will be excluded entirely ? | Thank you for raising this point. We have revised the manuscript to clarify the handling of mixed-age samples, as suggested. Studies that do not stratify by age will be excluded from the review.    See lines: 216-217  Trials where the data for people over 50 and under 50 is not reported separately will be excluded. |
| 3. Risk-of-bias tool  You plan to use the original Cochrane RoB tool (ROB-1), while ROB-2 is current standard. You note the decision was made for accessibility — which is reasonable — but some journals now expect ROB-2 unless strong justification exists.  Suggestion: explicitly acknowledging ROB-2 differences confirming that RoB domains retained adequately address randomisation & analysis issues, or stating whether ROB-2 may be piloted in parallel (even for a subset) | Many thanks for addressing this matter. As you have suggested we are choosing the original ROB tool for its accessibility, especially considering the public will be invited to assess parts of the risk of bias. We have now added additional statements within the manuscript to further justify this choice.    See lines 368-375 in *How will we check for bias in the studies? – risk of bias*    An important step in a systematic review is checking if there are any potential flaws (known as bias) in how the studies were done, how these flaws may have impacted the results, and therefore influence how much or how little we can trust the study. We call this risk of bias. To measure the trials’ risk of bias, we will use Cochrane’s original risk of bias tool (58). This tool looks at seven different aspects (called domains) of the randomised trial process for each outcome (i.e. quality of life or functional fitness) within the trial. While there has been an update to the Cochrane risk of bias assessment (ROB-2) tool (59) we will use the original version as it is easier to use than ROB-2 (60-63). The domains of the risk of bias tool are described in Table 1.    And see lines 661-670 in the *Discussion* section    A limitation of this review that may be considered is that we are not using the most up-to-date version of the Risk of Bias tool – ROB-2 (59). ROB-2 has been criticised as being overly complicated, requiring extensive training, and there has been limited implementation of the tool among systematic reviewers (60–63). Therefore, for this review we will use the original Risk of Bias tool (58) as it is sufficiently similar to the updated ROB-2 as it assesses the same domains, but is more accessible and easier to use, especially for people without experience. We will address one of the main limitations of the original tool by assessing each outcome (quality of life and heart health) instead of assessing the risk of bias for each study as a whole. Future reviews could consider using the updated tool (59) or ROBUST-RCT (104) another recently published tool for assessing risk of bias in randomised trials. |
| 4. Meta-analysis approach & heterogeneity  The plan appropriately uses random-effects inverse-variance meta-analysis, with good explanation of I² interpretation. However different instruments will likely be used for QoL and functional fitness, and SMD interpretation for citizen-scientists may be challenging  Suggestion: State how SMDs will be clinically interpreted (e.g., small/medium/large thresholds), and whether MID-anchored interpretation will be attempted for QoL tools (if available) | Thank you for your comment. Citizen scientists will not be asked to calculate or directly interpret SMD’s. All SMD’s will be calculated by the technical team. Using a random-effects, inverse variance approach the technical team will conduct the meta-analysis (if appropriate). Citizen scientists will instead interpret the resulting forest plots, with guidance and training. Regarding SMD-anchored interpretation we will follow GRADE Guidance 41 (Hulcrantz et al., 2025) which states that evidence synthesis authors may choose the thresholds of “no effect” for certainty assessment with targets of nonzero benefit and nonzero harm to avoid value judgement. Therefore, we will not be interpreting SMDs using small, medium or large thresholds.    For clarity we have added in some additional sentences throughout the manuscript outlining what the Technical Team and the People are doing throughout the synthesis.    Additionally, we have added in further detail to clarity that we will be using the threshold of ‘no effect’ in the GRADE assessment. See lines 577-587:    The information to be included in the summary of findings tables for each outcome includes:    •The number of trials and the number of participants included in the analysis.  •The estimated effect (measured using either the mean difference or the standardised mean difference) and 95% confidence interval. The effect estimate will be reported to either have or not have an effect in line with best practice guidelines (82). This is known as a threshold of “no effect”.  •The overall GRADE certainty of evidence rating (high, moderate, low or very low) with an explanation for this rating.  •Plain language summary explaining what the GRADE rating means. |
| 5. Citizen-science operational detail  The participation model is innovative and a key strength. However, practical safeguards should be clearer.  Suggestion — clarify the following  • Training provided to public screeners & extractors  • How data quality monitoring will occur  • How disagreements will be fed back as learning rather than correction  • Whether public contributors are blinded to intervention identity during RoB assessment (to minimise expectation bias)  These details will reassure readers that citizen-science contributes meaningfully without compromising methodological rigor. | Thank you for this comment. We have now added in a section in the Methods section ‘How will the People be supported to be involved in ‘doing’ the review?’ outlining the practical safeguards in place to support the public. We have also made further reference to our previous publication outlining this novel participatory approach in detail (Quinn et al., 2024).    See lines 178-188 for details.  **How will the People be supported to be involved in doing this review?**    The People's Review follows the principles of ‘learning by doing’ (38) and principles of citizen science (39). There are many tasks involved in a systematic review. To support the People to get involved in these tasks we will provide bespoke interactive training for each systematic review task including short animations, explanations of key concepts, and practice material. Support will also be provided in real time to the People while doing the task through pop-out reference material and hint features. As with all citizen science projects there are several quality control layers to ensure our review is accurate and high-quality including agreement algorithms and verification processes. We will provide regular updates via email, social media, newsletters and on our website to further support learning and maintain an open and honest feedback loop. Further description of these processes are described elsewhere (1).    Regarding blinding to intervention identity during ROB assessment – the public contributors will not be blinded to intervention identity during ROB assessment. This is not typically done in reviews and therefore we will not be doing this for our review. As with a ‘conventional’ review the ROB assessment will be done using the full-text study (and other documents if necessary) and it is up to the ROB assessor to determine the decision for each domain based on this information. The study will not be manipulated in anyway when presented to the public to assess the ROB. |
| 6. Subgroup analyses  Planned subgroup analyses (age bands, sex/gender, health status) are appropriate — but risk multiplicity and instability if few trials exist.  Suggestion: State that subgroup findings will be considered exploratory, analyses require adequate study numbers, and interaction testing will be preferred over stratified comparison where possible | Many thanks for flagging this matter. We have now added an additional sentence for further clarification on sub-group analysis to minimise the risk of multiplicity and instability.    See lines 542-543    The Technical Team will conduct a sub-group analysis only if there is sufficient data available in each sub-group (at least 2 studies) (75) and will be considered exploratory. |
| **Minor Comments** | |
| 1. Funding & competing interest statements  There appears to be repeated wording in the metadata text. You might remove duplication for clarity. | The funding and competing interest's statements have now been amended to remove duplication. |
| 2. Search strategy  Excellent database coverage including grey literature — good practice.  Consider also clarifying: who peer-reviewed the search (e.g., PRESS checklist used?) | Thank you for this comment. The search strategy was peer-reviewed by a medical librarian using the PRESS checklist. We have now added additional information for further clarification:    See line 262-263  The search strategy will be independently peer-reviewed by a medical librarian using the PRESS checklist (49). |
| 3. Definition of “≥8 sessions” Well-defined, but frequency (e.g., 2–3×/week) and duration per session may differ widely. State whether these were explored narratively. | Thank you for this comment. We will use ≥8 sessions for the eligibility criteria to guide the screening process regardless of frequency or duration.    We have added additional information to line 237-240 for further clarity on this matter:    We will include trials that compare resistance training with aerobic exercise where the programme includes at least eight sessions (as chosen by the People), regardless of how often (frequency) and how long (duration) each session was. Trials with fewer than eight sessions will not be included in the review.    However, as reported in section “*How will we collect the information from the studies? – data extraction*” lines 375-377, data on the frequency and duration of the intervention will be extracted and reported in the review. |
| 4. Meta-analysis software You used MetaAnalysisOnline.com. This is fine, but journals often prefer reproducible scripts (e.g., R, RevMan, Stata). If possible, retain an exportable dataset and log to document analytic steps. | Thank you for this comment – it has made us reflect on the detail (or lack thereof) within the protocol around data availability more generally. As such, we have now included a whole new section outlining how and where data will be shared.    Regarding MetaAnalysisOnline.com specifically, we have chosen this software as it, aligns with RevMan and offers similar functionality but is open-access and freely available and therefore aligns with the overall aim of The People's Review to make systematic reviews more open and accessible. While MetaAnalysisOnline.com does not create reproducible scripts. There is a function within the software to create permanent shareable links (albeit for 1 year only) and we will use this function and share it within the review manuscript and dataset. The data generated from this review will be shared on OSF also for openness, transparency and reproducibility.    See new section *How will we share data and results from our review?* lines 643-650    **How will we share data and results from our review?**  We will write-up and share the results of this review in plain English and submit it to a peer-reviewed, open-access journal so that the review is accessible to as many people as possible. We will share a pre-print of the review before it is submitted to the journal, so the results are available as soon as possible. We will also co-create with the People material to share the results in more accessible formats on our website, through social media and other channels. Additionally, we will share the data generated from this review freely and openly in OSF including a permanent shareable link to the meta-analysis conducted within MetaAnalysisOnline.com (74). |
