# Supplementary material for: Does resistance training make a difference to the quality of life or heart health for older adults compared to aerobic exercise? A systematic review protocol from The People’s Review
Source: PLoS One. 2026 Jun 10;21(6):e0337017. doi: 10.1371/journal.pone.0337017 (PMC13252789; doi:10.1371/journal.pone.0337017)
Supplement: Peer review history

# Peer review: Decision Letter 0

**DOI:** 10.1371/journal.pone.0337017.r001

**Authors:** Nishant Jaiswal

7 Jan 2026

PONE-D-25-58754

Does resistance training make a difference to the quality of life or heart health for older adults compared to aerobic exercise? A systematic review protocol from The People's Review

PLOS One

Dear Dr. Quinn,

Thank you for submitting your manuscript to PLOS ONE. After careful consideration, we feel that it has merit but does not fully meet PLOS ONE’s publication criteria as it currently stands. Therefore, we invite you to submit a revised version of the manuscript that addresses the points raised during the review process.

Please submit your revised manuscript by Feb 21 2026 11:59PM. If you will need more time than this to complete your revisions, please reply to this message or contact the journal office at plosone@plos.org. When you're ready to submit your revision, log on to https://www.editorialmanager.com/pone/ and select the 'Submissions Needing Revision' folder to locate your manuscript file.

Please include the following items when submitting your revised manuscript:

- A letter that responds to each point raised by the academic editor and reviewer(s). You should upload this letter as a separate file labeled 'Response to Reviewers'.
- A marked-up copy of your manuscript that highlights changes made to the original version. You should upload this as a separate file labeled 'Revised Manuscript with Track Changes'.
- An unmarked version of your revised paper without tracked changes. You should upload this as a separate file labeled 'Manuscript'.

If you would like to make changes to your financial disclosure, please include your updated statement in your cover letter. Guidelines for resubmitting your figure files are available below the reviewer comments at the end of this letter.

If applicable, we recommend that you deposit your laboratory protocols in protocols.io to enhance the reproducibility of your results. Protocols.io assigns your protocol its own identifier (DOI) so that it can be cited independently in the future. For instructions see: https://journals.plos.org/plosone/s/submission-guidelines#loc-laboratory-protocols. Additionally, PLOS ONE offers an option for publishing peer-reviewed Lab Protocol articles, which describe protocols hosted on protocols.io. Read more information on sharing protocols at https://plos.org/protocols?utm_medium=editorial-email&utm_source=authorletters&utm_campaign=protocols.

We look forward to receiving your revised manuscript.

Kind regards,

Nishant Premnath Jaiswal, MBBS, PhD

Academic Editor

PLOS One

Journal Requirements:

When submitting your revision, we need you to address these additional requirements.

1. Please ensure that your manuscript meets PLOS ONE's style requirements, including those for file naming. The PLOS ONE style templates can be found at

https://journals.plos.org/plosone/s/file?id=wjVg/PLOSOne_formatting_sample_main_body.pdf and

https://journals.plos.org/plosone/s/file?id=ba62/PLOSOne_formatting_sample_title_authors_affiliations.pdf

2.  We note that the grant information you provided in the ‘Funding Information’ and ‘Financial Disclosure’ sections do not match.

When you resubmit, please ensure that you provide the correct grant numbers for the awards you received for your study in the ‘Funding Information’ section.

3. One of the noted authors is a group or consortium “The People”. In addition to naming the author group, please list the individual authors and affiliations within this group in the acknowledgments section of your manuscript. Please also indicate clearly a lead author for this group along with a contact email address.

4. Your ethics statement should only appear in the Methods section of your manuscript. If your ethics statement is written in any section besides the Methods, please move it to the Methods section and delete it from any other section. Please ensure that your ethics statement is included in your manuscript, as the ethics statement entered into the online submission form will not be published alongside your manuscript.

5. If the reviewer comments include a recommendation to cite specific previously published works, please review and evaluate these publications to determine whether they are relevant and should be cited. There is no requirement to cite these works unless the editor has indicated otherwise.

[Note: HTML markup is below. Please do not edit.]

Reviewers' comments:

Reviewer's Responses to Questions

**Comments to the Author**

1. Does the manuscript provide a valid rationale for the proposed study, with clearly identified and justified research questions?

The research question outlined is expected to address a valid academic problem or topic and contribute to the base of knowledge in the field.

Reviewer #1: Yes

**********

2. Is the protocol technically sound and planned in a manner that will lead to a meaningful outcome and allow testing the stated hypotheses?

The manuscript should describe the methods in sufficient detail to prevent undisclosed flexibility in the experimental procedure or analysis pipeline, including sufficient outcome-neutral conditions (e.g. necessary controls, absence of floor or ceiling effects) to test the proposed hypotheses and a statistical power analysis where applicable. As there may be aspects of the methodology and analysis which can only be refined once the work is undertaken, authors should outline potential assumptions and explicitly describe what aspects of the proposed analyses, if any, are exploratory.

Reviewer #1: Partly

**********

3. Is the methodology feasible and described in sufficient detail to allow the work to be replicable?

Descriptions of methods and materials in the protocol should be reported in sufficient detail for another researcher to reproduce all experiments and analyses. The protocol should describe the appropriate controls, sample size calculations, and replication needed to ensure that the data are robust and reproducible.

Reviewer #1: Yes

**********

4. Have the authors described where all data underlying the findings will be made available when the study is complete?

The PLOS Data policy requires authors to make all data underlying the findings described in their manuscript fully available without restriction, with rare exception, at the time of publication. The data should be provided as part of the manuscript or its supporting information, or deposited to a public repository. For example, in addition to summary statistics, the data points behind means, medians and variance measures should be available. If there are restrictions on publicly sharing data—e.g. participant privacy or use of data from a third party—those must be specified.

Reviewer #1: Yes

**********

5. Is the manuscript presented in an intelligible fashion and written in standard English?

PLOS ONE does not copyedit accepted manuscripts, so the language in submitted articles must be clear, correct, and unambiguous. Any typographical or grammatical errors should be corrected at revision, so please note any specific errors here.

Reviewer #1: Yes

**********

6. Review Comments to the Author

Please use the space provided to explain your answers to the questions above and, if applicable, provide comments about issues authors must address before this protocol can be accepted for publication. You may also include additional comments for the author, including concerns about research or publication ethics.

You may also provide optional suggestions and comments to authors that they might find helpful in planning their study.

(Please upload your review as an attachment if it exceeds 20,000 characters)

Reviewer #1: This is a well-constructed, clearly written systematic review protocol with an innovative and commendable emphasis on citizen science and public involvement throughout all stages of the review. The plain-language style improves accessibility and transparency. The methodology largely aligns with Cochrane standards and PRISMA-P guidance, with appropriate attention to bias, heterogeneity, equity, and certainty of evidence. Below are major and minor comments intended to strengthen clarity, rigor, and reproducibility.

Major Comments

1. Scope and rationale — very well justified, but clarify outcome definitions

You clearly explain why comparing resistance vs aerobic training in adults ≥50 years matters. However, the decision to use functional fitness as the sole measure of “heart health” deserves further clarification:

• Functional fitness reflects overall capacity rather than cardiac function alone

• Other cardiac outcomes (e.g., BP, VO₂max, LV function, lipid markers) are clinically meaningful

Suggestion: Add a brief justification explaining why functional fitness was prioritised over physiologic cardiac endpoints (e.g., relevance to the public, feasibility, comparability).

2. Inclusion criteria — clarify mixed-age samples

You state trials including participants <50 years will be included only if over-50 data are reported separately. Many trials do not stratify by age.

Suggestion: State explicitly how such studies will be handled if stratified data are unavailable, whether authors will be contacted, and whether these trials will be excluded entirely ?

3. Risk-of-bias tool

You plan to use the original Cochrane RoB tool (ROB-1), while ROB-2 is current standard. You note the decision was made for accessibility — which is reasonable — but some journals now expect ROB-2 unless strong justification exists.

Suggestion: explicitly acknowledging ROB-2 differences confirming that RoB domains retained adequately address randomisation & analysis issues, or stating whether ROB-2 may be piloted in parallel (even for a subset)

4. Meta-analysis approach & heterogeneity

The plan appropriately uses random-effects inverse-variance meta-analysis, with good explanation of I² interpretation. However different instruments will likely be used for QoL and functional fitness, and SMD interpretation for citizen-scientists may be challenging

Suggestion: State how SMDs will be clinically interpreted (e.g., small/medium/large thresholds), and whether MID-anchored interpretation will be attempted for QoL tools (if available)

5. Citizen-science operational detail

The participation model is innovative and a key strength. However, practical safeguards should be clearer.

Suggestion — clarify the following

• Training provided to public screeners & extractors

• How data quality monitoring will occur

• How disagreements will be fed back as learning rather than correction

• Whether public contributors are blinded to intervention identity during RoB assessment (to minimise expectation bias)

These details will reassure readers that citizen-science contributes meaningfully without compromising methodological rigor.

6. Subgroup analyses

Planned subgroup analyses (age bands, sex/gender, health status) are appropriate — but risk multiplicity and instability if few trials exist.

Suggestion: State that subgroup findings will be considered exploratory, analyses require adequate study numbers, and interaction testing will be preferred over stratified comparison where possible

Minor Comments

1. Funding & competing interest statements

There appears to be repeated wording in the metadata text. You might remove duplication for clarity.

2. Search strategy

Excellent database coverage including grey literature — good practice.

Consider also clarifying: who peer-reviewed the search (e.g., PRESS checklist used?)

3. Definition of “≥8 sessions”

Well-defined, but frequency (e.g., 2–3×/week) and duration per session may differ widely. State whether these were explored narratively.

4. Meta-analysis software

You used MetaAnalysisOnline.com. This is fine, but journals often prefer reproducible scripts (e.g., R, RevMan, Stata). If possible, retain an exportable dataset and log to document analytic steps.

**********

7. PLOS authors have the option to publish the peer review history of their article (what does this mean?). If published, this will include your full peer review and any attached files.

If you choose “no”, your identity will remain anonymous but your review may still be made public.

**Do you want your identity to be public for this peer review?** For information about this choice, including consent withdrawal, please see our Privacy Policy.

Reviewer #1: No

**********

[NOTE: If reviewer comments were submitted as an attachment file, they will be attached to this email and accessible via the submission site. Please log into your account, locate the manuscript record, and check for the action link "View Attachments". If this link does not appear, there are no attachment files.]

To ensure your figures meet our technical requirements, please review our figure guidelines: https://journals.plos.org/plosone/s/figures

You may also use PLOS’s free figure tool, NAAS, to help you prepare publication quality figures: https://journals.plos.org/plosone/s/figures#loc-tools-for-figure-preparation.

NAAS will assess whether your figures meet our technical requirements by comparing each figure against our figure specifications.

# Author comment: Author response to Decision Letter 1

**DOI:** 10.1371/journal.pone.0337017.r002

23 Jan 2026

Dear Editor,

Many thanks for reviewing this manuscript - PONE-D-25-58754 - Does resistance training make a difference to the quality of life or heart health for older adults compared to aerobic exercise? A systematic review protocol from The People's Review. We are grateful for the time spent from the editorial office and peer-reviewers reviewing and sharing feedback on this manuscript. Please find below a line-by-line response to the editor and peer-reviewer's comments. We have updated the manuscript where appropriate also. Thank you for considering this manuscript.

All the best,

Éle Quinn 1,2,3 - on behalf of the authors of this manuscript.

Email: e.quinn34@universityofgalway.ie –

1Discipline of Occupational Therapy, School of Health Sciences, University of Galway, Galway, Ireland.

2Evidence Synthesis Ireland and Cochrane Ireland, University of Galway, Galway, Ireland.

3Centre for Health Research Methodology, School of Nursing and Midwifery, University of Galway, Galway, Ireland.

Line-by-line response below:

Journal Requirements:

1. Please ensure that your manuscript meets PLOS ONE's style requirements, including those for file naming. The PLOS ONE style templates can be found at

https://journals.plos.org/plosone/s/file?id=wjVg/PLOSOne_formatting_sample_main_body.pdf and

https://journals.plos.org/plosone/s/file?id=ba62/PLOSOne_formatting_sample_title_authors_affiliations.pdf

The manuscript has now been revised in line with PLOS ONE’s style requirements.

2. We note that the grant information you provided in the ‘Funding Information’ and ‘Financial Disclosure’ sections do not match.

When you resubmit, please ensure that you provide the correct grant numbers for the awards you received for your study in the ‘Funding Information’ section.

The Funding Information has now been updated to match the Financial Disclosure within the manuscript (lines 684-690). The following funders have been added to the Funding Information: the HSC Research and Development Division of the Public Health Agency (Northern Ireland), College of Medicine, Nursing and Health Sciences, University of Galway (Ireland) and Evidence Synthesis Ireland. No grant number is available for funding from these agencies.

3. One of the noted authors is a group or consortium “The People”. In addition to naming the author group, please list the individual authors and affiliations within this group in the acknowledgments section of your manuscript. Please also indicate clearly a lead author for this group along with a contact email address.

Thank you for raising this point. The lead author of this group is Ele Quinn and if someone wishes to contact the group they can email: thepeoplesreview@universityofgalway.ie.

In line with our ethics approval (2023.06.012), we did not collect individual affiliation data for members of this group, and we do not have consent to publicly disclose this information. Many contributors are members of the public and do not hold academic or institutional affiliations. For these reasons, we are unable to list the affiliations for the group in the Acknowledgments section. The individual authors names are listed in the Acknowledgments section. As per the journal requirements https://journals.plos.org/plosone/s/file?id=ba62/PLOSOne_formatting_sample_title_authors_affiliations.pdf it is not required for group authors affiliations to be listed.

4. Your ethics statement should only appear in the Methods section of your manuscript. If your ethics statement is written in any section besides the Methods, please move it to the Methods section and delete it from any other section. Please ensure that your ethics statement is included in your manuscript, as the ethics statement entered into the online submission form will not be published alongside your manuscript.

Many thanks for flagging this. The ethics statement is now included in the Methods section from line 169-171.

5. If the reviewer comments include a recommendation to cite specific previously published works, please review and evaluate these publications to determine whether they are relevant and should be cited. There is no requirement to cite these works unless the editor has indicated otherwise.

Thank you for this comment. Reviewer comments do not include a recommendation to cite specific previously published works.

Reviewer #1 Comments & author reponse

This is a well-constructed, clearly written systematic review protocol with an innovative and commendable emphasis on citizen science and public involvement throughout all stages of the review. The plain-language style improves accessibility and transparency. The methodology largely aligns with Cochrane standards and PRISMA-P guidance, with appropriate attention to bias, heterogeneity, equity, and certainty of evidence. Below are major and minor comments intended to strengthen clarity, rigor, and reproducibility.

Response: Many thanks for your considerate and useful comments. We are especially grateful for your recognition of the citizen science approach, the emphasis on public involvement, and the use of plain-language reporting to enhance accessibility and transparency.

We have carefully considered all major and minor comments and have revised the manuscript accordingly to strengthen clarity, methodological rigor, and reproducibility. Point-by-point responses to each comment are provided below, with corresponding changes tracked in the revised manuscript.

Major Comments:

1. Scope and rationale — very well justified, but clarify outcome definitions

You clearly explain why comparing resistance vs aerobic training in adults ≥50 years matters. However, the decision to use functional fitness as the sole measure of “heart health” deserves further clarification:

• Functional fitness reflects overall capacity rather than cardiac function alone

• Other cardiac outcomes (e.g., BP, VO₂max, LV function, lipid markers) are clinically meaningful

Suggestion: Add a brief justification explaining why functional fitness was prioritised over physiologic cardiac endpoints (e.g., relevance to the public, feasibility, comparability).

Response: Thank you for this helpful comment. We agree that heart health can be assessed in many ways. As suggested, we have updated the manuscript to provide additional clarification and context for the decision to use functional fitness as the sole measure of heart health in this review.

Additional information provided in lines 244-257:

"There are many outcomes we could use to measure heart health – blood pressure, resting heart rate, fat (or lipid) markers such as cholesterol, VO₂max (the maximum amount of oxygen the body can use during exercise) and many more. However, for this review we will use functional fitness as the measure for heart health. The People prioritised several heart health outcomes and selected functional fitness as the outcome to focus on. Functional fitness (or functional aerobic capacity) is a person’s ability to keep exercising over a sustained time period, at a level where they can still talk without becoming very breathless. It is one of the best measures of overall fitness and heart health as it evaluates all the body’s systems working together during exercise (49,50). We will include studies that measure functional fitness through accepted tests such as the 6-minute walk test (6MWT), shuttle tests or other methods."

2. Inclusion criteria — clarify mixed-age samples

You state trials including participants <50 years will be included only if over-50 data are reported separately. Many trials do not stratify by age.

Suggestion: State explicitly how such studies will be handled if stratified data are unavailable, whether authors will be contacted, and whether these trials will be excluded entirely ?

Response: Thank you for raising this point. We have revised the manuscript to clarify the handling of mixed-age samples, as suggested. Studies that do not stratify by age will be excluded from the review.

See lines: 216-217

Trials where the data for people over 50 and under 50 is not reported separately will be excluded.

3. Risk-of-bias tool

You plan to use the original Cochrane RoB tool (ROB-1), while ROB-2 is current standard. You note the decision was made for accessibility — which is reasonable — but some journals now expect ROB-2 unless strong justification exists.

Suggestion: explicitly acknowledging ROB-2 differences confirming that RoB domains retained adequately address randomisation & analysis issues, or stating whether ROB-2 may be piloted in parallel (even for a subset).

Response: Many thanks for addressing this matter. As you have suggested we are choosing the original ROB tool for its accessibility, especially considering the public will be invited to assess parts of the risk of bias. We have now added additional statements within the manuscript to further justify this choice.

See lines 368-375 in How will we check for bias in the studies? – risk of bias

An important step in a systematic review is checking if there are any potential flaws (known as bias) in how the studies were done, how these flaws may have impacted the results, and therefore influence how much or how little we can trust the study. We call this risk of bias. To measure the trials’ risk of bias, we will use Cochrane’s original risk of bias tool (58). This tool looks at seven different aspects (called domains) of the randomised trial process for each outcome (i.e. quality of life or functional fitness) within the trial. While there has been an update to the Cochrane risk of bias assessment (ROB-2) tool (59) we will use the original version as it is easier to use than ROB-2 (60-63). The domains of the risk of bias tool are described in Table 1.

And see lines 661-670 in the Discussion section

A limitation of this review that may be considered is that we are not using the most up-to-date version of the Risk of Bias tool – ROB-2 (59). ROB-2 has been criticised as being overly complicated, requiring extensive training, and there has been limited implementation of the tool among systematic reviewers (60–63). Therefore, for this review we will use the original Risk of Bias tool (58) as it is sufficiently similar to the updated ROB-2 as it assesses the same domains, but is more accessible and easier to use, especially for people without experience. We will address one of the main limitations of the original tool by assessing each outcome (quality of life and heart health) instead of assessing the risk of bias for each study as a whole. Future reviews could consider using the updated tool (59) or ROBUST-RCT (104) another recently published tool for assessing risk of bias in randomised trials.

4. Meta-analysis approach & heterogeneity

The plan appropriately uses random-effects inverse-variance meta-analysis, with good explanation of I² interpretation. However different instruments will likely be used for QoL and functional fitness, and SMD interpretation for citizen-scientists may be challenging

Suggestion: State how SMDs will be clinically interpreted (e.g., small/medium/large thresholds), and whether MID-anchored interpretation will be attempted for QoL tools (if available)

Response: Thank you for your comment. Citizen scientists will not be asked to calculate or directly interpret SMD’s. All SMD’s will be calculated by the technical team. Using a random-effects, inverse variance approach the technical team will conduct the meta-analysis (if appropriate). Citizen scientists will instead interpret the resulting forest plots, with guidance and training. Regarding SMD-anchored interpretation we will follow GRADE Guidance 41 (Hulcrantz et al., 2025) which states that evidence synthesis authors may choose the thresholds of “no effect” for certainty assessment with targets of nonzero benefit and nonzero harm to avoid value judgement. Therefore, we will not be interpreting SMDs using small, medium or large thresholds.

For clarity we have added in some additional sentences throughout the manuscript outlining what the Technical Team and the People are doing throughout the synthesis.

Additionally, we have added in further detail to clarity that we will be using the threshold of ‘no effect’ in the GRADE assessment. See lines 577-587:

The information to be included in the summary of findings tables for each outcome includes:

•The number of trials and the number of participants included in the analysis.

•The estimated effect (measured using either the mean difference or the standardised mean difference) and 95% confidence interval. The effect estimate will be reported to either have or not have an effect in line with best practice guidelines (82). This is known as a threshold of “no effect”.

•The overall GRADE certainty of evidence rating (high, moderate, low or very low) with an explanation for this rating.

•Plain language summary explaining what the GRADE rating means.

5. Citizen-science operational detail

The participation model is innovative and a key strength. However, practical safeguards should be clearer.

Suggestion — clarify the following

• Training provided to public screeners & extractors

• How data quality monitoring will occur

• How disagreements will be fed back as learning rather than correction

• Whether public contributors are blinded to intervention identity during RoB assessment (to minimise expectation bias)

These details will reassure readers that citizen-science contributes meaningfully without compromising methodological rigor.

Response: Thank you for this comment. We have now added in a section in the Methods section ‘How will the People be supported to be involved in ‘doing’ the review?’ outlining the practical safeguards in place to support the public. We have also made further reference to our previous publication outlining this novel participatory approach in detail (Quinn et al., 2024).

See lines 178-188 for details.

How will the People be supported to be involved in doing this review?

The People's Review follows the principles of ‘learning by doing’ (38) and principles of citizen science (39). There are many tasks involved in a systematic review. To support the People to get involved in these tasks we will provide bespoke interactive training for each systematic review task including short animations, explanations of key concepts, and practice material. Support will also be provided in real time to the People while doing the task through pop-out reference material and hint features. As with all citizen science projects there are several quality control layers to ensure our review is accurate and high-quality including agreement algorithms and verification processes. We will provide regular updates via email, social media, newsletters and on our website to further support learning and maintain an open and honest feedback loop. Further description of these processes are described elsewhere (1).

Regarding blinding to intervention identity during ROB assessment – the public contributors will not be blinded to intervention identity during ROB assessment. This is not typically done in reviews and therefore we will not be doing this for our review. As with a ‘conventional’ review the ROB assessment will be done using the full-text study (and other documents if necessary) and it is up to the ROB assessor to determine the decision for each domain based on this information. The study will not be manipulated in anyway when presented to the public to assess the ROB.

6. Subgroup analyses

Planned subgroup analyses (age bands, sex/gender, health status) are appropriate — but risk multiplicity and instability if few trials exist.

Suggestion: State that subgroup findings will be considered exploratory, analyses require adequate study numbers, and interaction testing will be preferred over stratified comparison where possible

Response: Many thanks for flagging this matter. We have now added an additional sentence for further clarification on sub-group analysis to minimise the risk of multiplicity and instability.

See lines 542-543

The Technical Team will conduct a sub-group analysis only if th

Attachment Submitted filename: Response to Reviewers.docx

# Peer review: Decision Letter 1

**DOI:** 10.1371/journal.pone.0337017.r003

**Authors:** Nishant Jaiswal

23 Feb 2026

PONE-D-25-58754R1

Does resistance training make a difference to the quality of life or heart health for older adults compared to aerobic exercise? A systematic review protocol from The People's Review

PLOS One

Dear Dr. Quinn,

Thank you for submitting your manuscript to PLOS ONE. After careful consideration, we feel that it has merit but does not fully meet PLOS ONE’s publication criteria as it currently stands. Therefore, we invite you to submit a revised version of the manuscript that addresses the points raised during the review process.

Please submit your revised manuscript by Apr 09 2026 11:59PM. If you will need more time than this to complete your revisions, please reply to this message or contact the journal office at plosone@plos.org. When you're ready to submit your revision, log on to https://www.editorialmanager.com/pone/ and select the 'Submissions Needing Revision' folder to locate your manuscript file.

Please include the following items when submitting your revised manuscript:

- A letter that responds to each point raised by the academic editor and reviewer(s). You should upload this letter as a separate file labeled 'Response to Reviewers'.
- A marked-up copy of your manuscript that highlights changes made to the original version. You should upload this as a separate file labeled 'Revised Manuscript with Track Changes'.
- An unmarked version of your revised paper without tracked changes. You should upload this as a separate file labeled 'Manuscript'.

If you would like to make changes to your financial disclosure, please include your updated statement in your cover letter. Guidelines for resubmitting your figure files are available below the reviewer comments at the end of this letter.

If applicable, we recommend that you deposit your laboratory protocols in protocols.io to enhance the reproducibility of your results. Protocols.io assigns your protocol its own identifier (DOI) so that it can be cited independently in the future. For instructions see: https://journals.plos.org/plosone/s/submission-guidelines#loc-laboratory-protocols. Additionally, PLOS ONE offers an option for publishing peer-reviewed Lab Protocol articles, which describe protocols hosted on protocols.io. Read more information on sharing protocols at https://plos.org/protocols?utm_medium=editorial-email&utm_source=authorletters&utm_campaign=protocols.

We look forward to receiving your revised manuscript.

Kind regards,

Nishant Premnath Jaiswal, MBBS, PhD

Academic Editor

PLOS One

**Journal Requirements:**

If the reviewer comments include a recommendation to cite specific previously published works, please review and evaluate these publications to determine whether they are relevant and should be cited. There is no requirement to cite these works unless the editor has indicated otherwise.

[Note: HTML markup is below. Please do not edit.]

Reviewers' comments:

Reviewer's Responses to Questions

**Comments to the Author**

1. Does the manuscript provide a valid rationale for the proposed study, with clearly identified and justified research questions?

The research question outlined is expected to address a valid academic problem or topic and contribute to the base of knowledge in the field.

Reviewer #1: Yes

**********

2. Is the protocol technically sound and planned in a manner that will lead to a meaningful outcome and allow testing the stated hypotheses?

The manuscript should describe the methods in sufficient detail to prevent undisclosed flexibility in the experimental procedure or analysis pipeline, including sufficient outcome-neutral conditions (e.g. necessary controls, absence of floor or ceiling effects) to test the proposed hypotheses and a statistical power analysis where applicable. As there may be aspects of the methodology and analysis which can only be refined once the work is undertaken, authors should outline potential assumptions and explicitly describe what aspects of the proposed analyses, if any, are exploratory.

Reviewer #1: Partly

**********

3. Is the methodology feasible and described in sufficient detail to allow the work to be replicable?

Descriptions of methods and materials in the protocol should be reported in sufficient detail for another researcher to reproduce all experiments and analyses. The protocol should describe the appropriate controls, sample size calculations, and replication needed to ensure that the data are robust and reproducible.

Reviewer #1: No

**********

4. Have the authors described where all data underlying the findings will be made available when the study is complete?

The PLOS Data policy requires authors to make all data underlying the findings described in their manuscript fully available without restriction, with rare exception, at the time of publication. The data should be provided as part of the manuscript or its supporting information, or deposited to a public repository. For example, in addition to summary statistics, the data points behind means, medians and variance measures should be available. If there are restrictions on publicly sharing data—e.g. participant privacy or use of data from a third party—those must be specified.

Reviewer #1: No

**********

5. Is the manuscript presented in an intelligible fashion and written in standard English?

PLOS ONE does not copyedit accepted manuscripts, so the language in submitted articles must be clear, correct, and unambiguous. Any typographical or grammatical errors should be corrected at revision, so please note any specific errors here.

Reviewer #1: Yes

**********

6. Review Comments to the Author

Please use the space provided to explain your answers to the questions above and, if applicable, provide comments about issues authors must address before this protocol can be accepted for publication. You may also include additional comments for the author, including concerns about research or publication ethics.

You may also provide optional suggestions and comments to authors that they might find helpful in planning their study.

(Please upload your review as an attachment if it exceeds 20,000 characters)

Reviewer #1: Dear authors

I have carefully reviewed the protocol in full. Below are detailed, structured comments focusing on scientific rigor, clarity, methodology, reporting standards, and publishability.

Major Comments

1. Conceptual Framing of “Heart Health”

You define heart health exclusively as functional fitness (e.g., 6MWT).

Strengths

• Clear justification based on public prioritisation.

• Transparent acknowledgement that other cardiac outcomes exist.

Concerns

• Functional fitness is not a direct measure of cardiac physiology.

• It may reflect musculoskeletal, pulmonary, motivational, and neurological contributions.

• The conceptual shift from “heart health” to “functional aerobic capacity” could be misinterpreted.

Recommendation

Strengthen internal consistency by:

• Explicitly stating that heart health is operationalised as functional fitness for this review.

• Adding one clarifying sentence noting that functional fitness is a proxy measure rather than a direct cardiac biomarker.

This will protect against criticism from cardiology-focused reviewers.

2. Restriction to ≥8 Sessions

The eligibility criterion of ≥8 sessions is clearly justified as public-chosen.

However:

• Why 8 sessions specifically?

• Is there empirical precedent?

• Could this exclude short but physiologically meaningful RCTs?

Recommendation

Add one brief methodological justification such as:

• Eight sessions approximates a minimal exposure threshold for physiological adaptation.

• Or clarify that this threshold balances feasibility and meaningful intervention dose.

Without rationale, this may appear arbitrary.

3. Risk of Bias Tool (ROB-1 vs ROB-2)

Your justification for using ROB-1 is thoughtful and transparent.

However:

• ROB-1 uses "unclear risk"

• Your manuscript uses "some concerns" (ROB-2 terminology)

This may create conceptual inconsistency.

Recommendation

Ensure terminology matches ROB-1 exactly:

• Low risk

• High risk

• Unclear risk

Or clearly state if modified terminology is used.

Also consider adding:

“The domains assessed align conceptually with ROB-2, though structured differently.”

This will reassure methodologists.

4. Citizen Science Model – Methodological Safeguards

This is the most innovative aspect of your review.

You describe:

• Training modules

• 4-person extraction

• 80% agreement threshold

• Technical resolver

This is strong.

However, two clarifications would strengthen rigor:

A. Agreement Algorithm

How is 80% calculated?

• Per data item?

• Per study?

• Per domain?

Clarify the unit of agreement.

B. Bias Risk in Public Assessment

Public contributors assess first 4 ROB domains.

Potential issues:

• Understanding allocation concealment

• Understanding sequence generation

You might briefly clarify:

• Whether calibration exercises will be conducted.

• Whether example worked cases are provided.

These additions would enhance credibility.

5. Meta-Analysis and SMD Interpretation

You appropriately avoid Cohen’s small/medium/large thresholds.

This aligns with contemporary GRADE thinking.

However, for readers:

• If SMD is 0.25 or 0.40, how will this be interpreted narratively?

Recommendation:

Add one sentence clarifying that:

Effect sizes will be interpreted relative to the threshold of no effect and contextualised clinically where possible.

This prevents the impression that magnitude interpretation is avoided entirely.

6. Handling of Cluster and Crossover Trials

Your approach is appropriate.

However, clarify:

• For cluster trials: will ICC be extracted or assumed?

• If ICC not reported, will external ICC estimates be used?

A single clarifying sentence would strengthen analytic transparency.

7. Heterogeneity Interpretation

The I² table is clearly presented.

However:

• Overlap in thresholds is acknowledged, which is good.

• Consider adding Tau² (between-study variance) as a secondary descriptor.

Even if not emphasised publicly, noting it strengthens statistical rigor.

8. Reporting Bias and Funnel Plot

Appropriate threshold of >10 studies.

Clarify:

• If fewer than 10 studies, reporting bias will be discussed qualitatively.

This prevents methodological gaps.

9. Data Availability Statement

You now include OSF sharing and permanent links.

Strong transparency.

One suggestion:

Specify whether:

• Extracted dataset will be shared

• Risk of bias assessments will be shared

• Agreement metrics will be shared

That would elevate open science quality.

Minor Comments

1. Terminology Consistency

Occasional variation between:

“Older adults”

“People over 50”

“Adults over 50”

Choose one primary term and maintain consistency.

2. Quality of Life Instruments

You mention SF-36, EuroQol, WHOQOL.

Consider clarifying:

• Whether mental and physical component scores will be analysed separately if reported.

3. Gender vs Sex Data Extraction

You list:

• sex

• gender

• sexual identity

Strong equity inclusion.

Clarify whether:

• Analyses will distinguish sex vs gender where reported.

4. Language

• The plain English style is excellent and consistent.

• However, a few places could be slightly tightened for conciseness without losing accessibility.

• For example:

• “We are all one large review team…”

• Could be shortened slightly to improve flow.

5. Figures

Ensure that:

• Fig. 1 flow diagram

• Fig. 2 forest plot explanation

• Fig. 3 heterogeneity explanation

Are formatted according to PLOS ONE graphical standards.

**********

7. PLOS authors have the option to publish the peer review history of their article (what does this mean?). If published, this will include your full peer review and any attached files.

If you choose “no”, your identity will remain anonymous but your review may still be made public.

**Do you want your identity to be public for this peer review?** For information about this choice, including consent withdrawal, please see our Privacy Policy.

Reviewer #1: No

**********

[NOTE: If reviewer comments were submitted as an attachment file, they will be attached to this email and accessible via the submission site. Please log into your account, locate the manuscript record, and check for the action link "View Attachments". If this link does not appear, there are no attachment files.]

To ensure your figures meet our technical requirements, please review our figure guidelines: https://journals.plos.org/plosone/s/figures

You may also use PLOS’s free figure tool, NAAS, to help you prepare publication quality figures: https://journals.plos.org/plosone/s/figures#loc-tools-for-figure-preparation.

NAAS will assess whether your figures meet our technical requirements by comparing each figure against our figure specifications.

# Author comment: Author response to Decision Letter 2

**DOI:** 10.1371/journal.pone.0337017.r004

11 Mar 2026

Peer-reviewer major comments

1. Conceptual Framing of “Heart Health”

You define heart health exclusively as functional fitness (e.g., 6MWT).

Strengths

• Clear justification based on public prioritisation.

• Transparent acknowledgement that other cardiac outcomes exist.

Concerns

• Functional fitness is not a direct measure of cardiac physiology.

• It may reflect musculoskeletal, pulmonary, motivational, and neurological contributions.

• The conceptual shift from “heart health” to “functional aerobic capacity” could be misinterpreted.

Recommendation

Strengthen internal consistency by:

• Explicitly stating that heart health is operationalised as functional fitness for this review.

• Adding one clarifying sentence noting that functional fitness is a proxy measure rather than a direct cardiac biomarker.

This will protect against criticism from cardiology-focused reviewers.

Author response:

Thank you for this helpful comment. We agree that functional fitness is not a direct physiological measure of cardiac function. To improve clarity of this and to reduce the risk of misinterpretation, we have revised parts of the Outcomes section.

See lines 242-264:

“There are many outcomes we could use to measure heart health – blood pressure, resting heart rate, fat (or lipid) markers such as cholesterol, VO₂max (the maximum amount of oxygen the body can use during exercise) and many more. However, for this review we will use functional fitness as the measure for heart health. The People prioritised several heart health outcomes and selected functional fitness as the outcome to focus on. Functional fitness (or functional aerobic capacity) is a person’s ability to keep exercising over a sustained time period, at a level where they can still talk without becoming very breathless. It is one of the best measures of overall fitness and heart health as it evaluates all the body’s systems working together during exercise (49,50). While functional fitness does not directly measure heart function through biological markers like blood pressure and cholesterol levels, it is a valuable measure of overall heart health. We will include studies that measure functional fitness through accepted tests such as the 6-minute walk test (6MWT), shuttle tests or other methods.

No other outcomes will be explored in this review. We acknowledge that this review could focus on many other outcomes related to heart health. For this review we are focusing on functional fitness only as the measure of heart health. The People decided to focus only on functional fitness, as this was considered the most important measure of heart health for them.”

2. Restriction to ≥8 Sessions

The eligibility criterion of ≥8 sessions is clearly justified as public-chosen.

However:

• Why 8 sessions specifically?

• Is there empirical precedent?

• Could this exclude short but physiologically meaningful RCTs?

Recommendation

Add one brief methodological justification such as:

• Eight sessions approximates a minimal exposure threshold for physiological adaptation.

• Or clarify that this threshold balances feasibility and meaningful intervention dose.

Without rationale, this may appear arbitrary.

Author Response:

Thank you for raising this point. We have now added additional justification for the ≥8-session threshold in the eligibility criteria. This threshold was selected the public to and reflects a balance between meaningful exercise dose while remaining feasible in most settings. The manuscript has been revised for clarification. See lines 227-232.

“We will include trials that compare resistance training with aerobic exercise where the programme includes at least eight sessions, regardless of how often (frequency) and how long (duration) each session was. The People chose this threshold because it provides enough time for participants to receive enough exercise for their bodies to adapt and show changes in fitness, while still being feasible to carry out in real-world settings. Trials with fewer than eight sessions will be excluded from this review.”

3. Risk of Bias Tool (ROB-1 vs ROB-2)

Your justification for using ROB-1 is thoughtful and transparent.

However:

• ROB-1 uses "unclear risk"

• Your manuscript uses "some concerns" (ROB-2 terminology)

This may create conceptual inconsistency.

Recommendation

Ensure terminology matches ROB-1 exactly:

• Low risk

• High risk

• Unclear risk

Or clearly state if modified terminology is used.

Also consider adding:

“The domains assessed align conceptually with ROB-2, though structured differently.”

This will reassure methodologists.

Author Response:

Thank you for highlighting this inconsistency. We have now amended the terminology throughout the manuscript to align fully with the ROB-1 tool.

See line 381:

“For each domain, we will assign a judgement of either low, high or unclear risk of bias.”

Additionally, we have added in additional information in the Limitations section to highlight that the ROB-2 is conceptually similar to ROB though structured differently.

See lines 672-682:

“A limitation of this review that may be considered is that we are not using the most up-to-date version of the Risk of Bias tool – ROB-2 (59). ROB-2 has been criticised as being overly complicated, requiring extensive training, and there has been limited implementation of the tool among systematic reviewers (60–63). Therefore, for this review we will use the original Risk of Bias tool (58) as it is similar to the updated ROB-2 as it assesses the same domains, but is structured differently and in a more accessible and easier to use way - especially for people without experience. We will address one of the main limitations of the original tool by assessing each outcome (quality of life and heart health) instead of assessing the risk of bias for each study as a whole. Future reviews could consider using the updated tool (59) or ROBUST-RCT (104) another recently published tool for assessing risk of bias in randomised trials.”

4. Citizen Science Model – Methodological Safeguards

This is the most innovative aspect of your review.

You describe:

• Training modules

• 4-person extraction

• 80% agreement threshold

• Technical resolver

This is strong.

However, two clarifications would strengthen rigor:

A. Agreement Algorithm

How is 80% calculated?

• Per data item?

• Per study?

• Per domain?

Clarify the unit of agreement.

B. Bias Risk in Public Assessment

Public contributors assess first 4 ROB domains.

Potential issues:

• Understanding allocation concealment

• Understanding sequence generation

You might briefly clarify:

• Whether calibration exercises will be conducted.

• Whether example worked cases are provided.

These additions would enhance credibility.

Author Response:

Thank you for this helpful feedback on the citizen science model. As suggested, we have revised the manuscript to clarify both the agreement algorithm and the training procedures used to support the public.

We specify that, for data extraction, the 80% agreement threshold is calculated per data item. If fewer than 80% of contributors extract the same value for a given item, the discrepancy is flagged and resolved by a member of the Technical Team. See lines 348-354 for details.

“People may extract slightly different information from each study. When there is less than 80% agreement for each data item extracted by the People, a resolver from the Technical Team will make the final decision. If the Technical Team members do not agree, this disagreement will be resolved through discussion or involving a third team member if necessary.”

For the risk of bias assessment, the 80% agreement threshold is applied per domain. Each study will be assessed independently by at least four public contributors. Where agreement falls below the 80% threshold, the Technical Team will review the judgement and make the final decision. See lines 387-392.

“The People will look at the first four risk of bias domains, with the remaining three domains to be assessed independently by two members of the Technical Team. Each study will be assessed by at least four different members of the public. If there is less than 80% agreement for each domain assessed by the People, a member of the Technical Team will act as a resolver to make the final decision. If discrepancies arise in the Technical Team, they will resolve it through discussion or with a third assessor.”

We have also expanded the description of training and calibration procedures to clarify that public contributors will complete structured training modules, worked examples, and practice exercises before undertaking risk-of-bias assessments (lines 381-385).

“For each domain, we will assign a judgement of either low, high or unclear risk of bias. As with previous stages, bespoke training will be available to explain key concepts about risk of bias, provide worked examples and allow people to practice before assessing the risk of bias in the included studies. The public can assess the risk of bias in as many studies as they choose; however, they will be asked to assess the risk of bias in at least one study.”

5. Meta-Analysis and SMD Interpretation

You appropriately avoid Cohen’s small/medium/large thresholds.

This aligns with contemporary GRADE thinking.

However, for readers:

• If SMD is 0.25 or 0.40, how will this be interpreted narratively?

Recommendation:

Add one sentence clarifying that:

Effect sizes will be interpreted relative to the threshold of no effect and contextualised clinically where possible.

This prevents the impression that magnitude interpretation is avoided entirely.

Author Response:

Thank you for raising this point. In line with recent GRADE guidance (Hultcrantz et al., 2025), we will interpret effect estimates relative to the threshold of no effect rather than using minimally important difference. To clarify this approach, we have added additional text explaining how effect estimates and confidence intervals will be interpreted using GRADE (lines 588–600).

“GRADE results of a systematic review are usually presented in a table called “summary of findings”, to make it easier for the user to understand the overall review results quickly. The information to be included in the summary of findings tables for each outcome includes:

• The number of trials and the number of participants included in the analysis.

• The estimated effect (measured using either the mean difference or the standardised mean difference) and 95% confidence interval. The effect estimate will be reported to either have or not have an effect in line with best practice guidelines (82). This is known as a threshold of “no effect”.

• The overall GRADE certainty of evidence rating (high, moderate, low or very low) with an explanation for this rating.

• Plain language summary explaining what the GRADE rating means and contextualising this to everyday health decision making if possible.”

6. Handling of Cluster and Crossover Trials

Your approach is appropriate.

However, clarify:

• For cluster trials: will ICC be extracted or assumed?

• If ICC not reported, will external ICC estimates be used?

A single clarifying sentence would strengthen analytic transparency.

Author Response:

Thank you for this useful suggestion. We have added further detail to Table 2 describing how cluster-randomised trials will be handled in this review – specifically addressing the intracluster correlation coefficient (ICC).

See Table 2. Study designs that need to be accounted in this review for details:

“The Technical Team will assess if the authors of the cluster trials have appropriately accounted for clustering and get advice from a statistician if needed. If the authors have not appropriately accounted for clusters, we will adjust for this using methods recommended in the Cochrane handbook. These methods require specific data (such as the intracluster correlation coefficient (ICC)) to make appropriate adjustments. If this is not reported in the study we will contact the study authors or use external estimates and clearly document the source used (66).”

7. Heterogeneity Interpretation

The I² table is clearly presented.

However:

• Overlap in thresholds is acknowledged, which is good.

• Consider adding Tau² (between-study variance) as a secondary descriptor.

Even if not emphasised publicly, noting it strengthens statistical rigor.

Author Response:

Thank you for this suggestion. We have now added Tau² (between study variance) as an additional measure of heterogeneity. See lines 493-496 for details.

“We will also complete a statistical test called Tau-squared. Tau-squared measures variance between studies in a meta-analysis. Tau-squared is presented as a number with larger values indicating more variation between studies, and therefore more heterogeneity (69).”

8. Reporting Bias and Funnel Plot

Appropriate threshold of >10 studies.

Clarify:

• If fewer than 10 studies, reporting bias will be discussed qualitatively.

This prevents methodological gaps.

Authors Response:

Thank you for highlighting this point. We have added text clarifying that when fewer than 10 studies are available. See lines 518-521.

“If there are fewer than 10 studies available funnel plots are unreliable, so we will search trial registries for unpublished studies. We will also compare published study reports with pre-determined plans (for example trial registry information, protocols or statistical analysis plans).”

9. Data Availability Statement

You now include OSF sharing and permanent links.

Strong transparency.

One suggestion:

Specify whether:

• Extracted dataset will be shared

• Risk of bias assessments will be shared

• Agreement metrics will be shared

That would elevate open science quality.

Author Response:

We have now included additional information in the data availability statement to clarify that extracted data, risk of bias assessments and results from agreement metrics will be published once the review is complete. See data availability statement below:

“Data collected during this review process including extracted data, risk of bias assessment and decisions from agreement algorithms, will be publicly available when the review is completed and published.”

Peer-review Minor Comments

1. Terminology Consistency

Occasional variation between:

“Older adults”

“People over 50”

“Adults over 50”

Choose one primary term and maintain consistency.

Author Response:

Thank you for flagging this. We have now amended the manuscript to use the term older adults consistently throughout and explained in the eligibility criteria that for this review we use the threshold of over 50 as our definition of older adult.

2. Quality of Life Instruments

You mention SF-36, EuroQol, WHOQOL.

Consider clarifying:

• Whether mental and physical component scores will be analysed separately if reported.

Author Response:

Thank you for highlighting this point. We have clarified that where quality-of-life instruments report separate mental and physical component scores, these domains will be extracted and analysed separately where appropriate. See lines 244-246 for details.

“These measures often include different areas including both the mental and physical aspects of quality of life. Both mental and physical health aspects will be collected and analysed separately, if available.”

3. Gender vs Sex Data Extraction

You list:

• sex

• gender

• sexual identity

Strong equity inclusion.

Clarify whether:

• Analyses will distinguish sex vs gender where reported.

Author Response:

Thank you for raising this. Sex and gender will be distinguished where reported, and this information will be presented in a participant characteristics table in line with PRO-EDI guidance. This is outlined in the data extraction and health equity sections.

4. Language

• The plain English style is excellent and consistent.

• However, a few places could be slightly tightened for concise

Attachment Submitted filename: Response_to_Reviewers.pdf

# Editor report: Decision Letter 2

**DOI:** 10.1371/journal.pone.0337017.r005

**Authors:** Nishant Jaiswal

1 May 2026

Does resistance training make a difference to the quality of life or heart health for older adults compared to aerobic exercise? A systematic review protocol from The People's Review

PONE-D-25-58754R2

Dear Dr. Quinn,

We’re pleased to inform you that your manuscript has been judged scientifically suitable for publication and will be formally accepted for publication once it meets all outstanding technical requirements.

Within one week, you’ll receive an e-mail detailing the required amendments. When these have been addressed, you’ll receive a formal acceptance letter and your manuscript will be scheduled for publication.

An invoice will be generated when your article is formally accepted. Please note, if your institution has a publishing partnership with PLOS and your article meets the relevant criteria, all or part of your publication costs will be covered. Please make sure your user information is up-to-date by logging into Editorial Manager at Editorial Manager® and clicking the ‘Update My Information' link at the top of the page. For questions related to billing, please contact billing support.

If your institution or institutions have a press office, please notify them about your upcoming paper to help maximize its impact. If they’ll be preparing press materials, please inform our press team as soon as possible -- no later than 48 hours after receiving the formal acceptance. Your manuscript will remain under strict press embargo until 2 pm Eastern Time on the date of publication. For more information, please contact onepress@plos.org.

Kind regards,

Nishant Premnath Jaiswal, MBBS, PhD

Academic Editor

PLOS One

Additional Editor Comments (optional):

I am satisfied with the revision and author's responses

Reviewers' comments:

# Editor report: Acceptance letter

**DOI:** 10.1371/journal.pone.0337017.r006

**Authors:** Nishant Jaiswal

PONE-D-25-58754R2

PLOS One

Dear Dr. Quinn,

I'm pleased to inform you that your manuscript has been deemed suitable for publication in PLOS One. Congratulations! Your manuscript is now being handed over to our production team.

At this stage, our production department will prepare your paper for publication. This includes ensuring the following:

* All references, tables, and figures are properly cited

* All relevant supporting information is included in the manuscript submission,

* There are no issues that prevent the paper from being properly typeset

You will receive further instructions from the production team, including instructions on how to review your proof when it is ready. Please keep in mind that we are working through a large volume of accepted articles, so please give us a few days to review your paper and let you know the next and final steps.

Lastly, if your institution or institutions have a press office, please let them know about your upcoming paper now to help maximize its impact. If they'll be preparing press materials, please inform our press team within the next 48 hours. Your manuscript will remain under strict press embargo until 2 pm Eastern Time on the date of publication. For more information, please contact onepress@plos.org.

You will receive an invoice from PLOS for your publication fee after your manuscript has reached the completed accept phase. If you receive an email requesting payment before acceptance or for any other service, this may be a phishing scheme. Learn how to identify phishing emails and protect your accounts at https://explore.plos.org/phishing.

If we can help with anything else, please email us at customercare@plos.org.

Thank you for submitting your work to PLOS ONE and supporting open access.

Kind regards,

PLOS ONE Editorial Office Staff

on behalf of

Dr. Nishant Premnath Jaiswal

Academic Editor

PLOS One
